# Pharmacological Rescue with SR8278, a Circadian Nuclear Receptor REV-ERBα Antagonist as a Therapy for Mood Disorders in Parkinson’s Disease

**DOI:** 10.1007/s13311-022-01215-w

**Published:** 2022-03-23

**Authors:** Jeongah Kim, Inah Park, Sangwon Jang, Mijung Choi, Doyeon Kim, Woong Sun, Youngshik Choe, Ji-Woong Choi, Cheil Moon, Sung Ho Park, Han Kyoung Choe, Kyungjin Kim

**Affiliations:** 1 Department of Brain Sciences, Daegu Gyeongbuk Institute of Science and Technology (DGIST), Daegu, 42988 Korea; 2 Department of Anatomy, College of Medicine, Korea University, Seoul, Korea; 3 Program in Neurosciences & Mental Health, The Hospital for Sick Children, Toronto, Ontario Canada; 4 Korea Brain Research Institute (KBRI), Daegu, Korea; 5 Department of Electrical Engineering and Computer Science, DGIST, Daegu, Korea; 6 Convergence Research Advanced Centre for Olfaction, DGIST, Daegu, Korea; 7 Department of Biological Sciences, Ulsan National Institute of Science and Technology (UNIST), Ulsan, 44919 Korea

**Keywords:** Parkinson’s disease, Dopaminergic neuronal loss, Sundowning syndrome, Circadian mood regulation, Rev-erbα, Nurr1

## Abstract

Parkinson’s disease is a neurodegenerative disease characterized by progressive dopaminergic neuronal loss. Motor deficits experienced by patients with Parkinson’s disease are well documented, but non-motor symptoms, including mood disorders associated with circadian disturbances, are also frequent features. One common phenomenon is “sundowning syndrome,” which is characterized by the occurrence of neuropsychiatric symptoms at a specific time (dusk), causing severe quality of life challenges. This study aimed to elucidate the underlying mechanisms of sundowning syndrome in Parkinson’s disease and their molecular links with the circadian clock. We demonstrated that 6-hydroxydopamine (6-OHDA)-lesioned mice, as Parkinson’s disease mouse model, exhibit increased depression- and anxiety-like behaviors only at dawn (the equivalent of dusk in human). Administration of REV-ERBα antagonist, SR8278, exerted antidepressant and anxiolytic effects in a circadian time-dependent manner in 6-OHDA-lesioned mice and restored the circadian rhythm of mood-related behaviors. 6-OHDA-lesion altered DAergic-specific *Rev-erbα* and *Nurr1* transcription, and atypical binding activities of REV-ERBα and NURR1, which are upstream nuclear receptors for the discrete tyrosine hydroxylase promoter region. SR8278 treatment restored the binding activities of REV-ERBα and NURR1 to the tyrosine hydroxylase promoter and the induction of enrichment of the R/N motif, recognized by REV-ERBα and NURR1, as revealed by ATAC-sequencing; therefore, tyrosine hydroxylase expression was elevated in the ventral tegmental area of 6-OHDA-injected mice, especially at dawn. These results indicate that REV-ERBα is a potential therapeutic target, and its antagonist, SR8278, is a potential drug for mood disorders related to circadian disturbances, namely sundowning syndrome, in Parkinson’s disease.

**Supplementary Information:**

The online version contains supplementary material available at 10.1007/s13311-022-01215-w.

## Introduction

Parkinson’s disease (PD) is a common neurodegenerative disorder characterized by the progressive degeneration of dopaminergic (DAergic) neurons in the substantia nigra pars compacta (SNpc). Patients with PD experience motor symptoms, such as tremor, rigidity, bradykinesia, and postural instability; however, non-motor symptoms, such as sleep disorders, psychiatric disorders, sensory disturbances, and autonomic disabilities [1, 2], are also common. Approximately 45% of patients with PD experience depression and anxiety [1, 3]. Disturbances in sleep–wake cycles and mood disorders are associated with dysregulated circadian rhythms [2, 4]. One relatively common circadian rhythm disturbance among elderly individuals and patients with dementia and neurodegenerative disorders, including PD, is “sundowning syndrome” [5]. This syndrome, also called nocturnal delirium, is characterized by the occurrence or exacerbation of behavioral disturbances, especially neuropsychiatric symptoms such as agitation, confusion, anxiety, and depression, in a temporal pattern during the late afternoon or early evening [4–6]. These behaviors disrupt both patients and caregivers, and circadian disturbances represent a serious detriment to an individual’s quality of life [5]. However, the neural basis underlying sundowning syndrome behavior and its connection with circadian rhythm disturbances remain unknown.

The notion that physiological and behavioral processes in mammals are regulated by circadian rhythms, driven by circadian clocks, has been widely accepted. The mammalian circadian clock is an endogenous time-keeping system composed of transcriptional-translational feedback loops [7]. The circadian system is organized in a hierarchical manner, with the master clock located in the hypothalamic suprachiasmatic nucleus (SCN) of the anterior hypothalamus, and local clocks located in both extra-SCN brain regions and peripheral organs [8, 9]. In fact, patients with mood disorders exhibit disrupted circadian rhythmicity in body temperature, hormone secretions (e.g., cortisol and melatonin), blood pressure, and sleep–wake cycles [10, 11]. Human and animal studies have shown molecular links between circadian rhythms and mood disorders [12].

Recently, we identified a functional link between mood regulation and the circadian timing system in midbrain DAergic neurons [13]. REV-ERBα, a circadian nuclear receptor encoded by the NR1D1 gene, was initially found to repress BMAL1 expression by competing with transcriptional activators (RORα/β/γ) [14, 15]. Notably, *Rev-erbα*-deficient mice had altered mood-related behaviors and a hyperdopaminergic state. REV-ERBα directly represses the expression of tyrosine hydroxylase (TH), which is a rate-limiting enzyme for DA biosynthesis, by competing with the nuclear receptor-related 1 protein (NURR1; an essential nuclear receptor for DAergic neuronal function), leading to the circadian rhythmicity of midbrain DAergic neurons. In fact, there are cis-acting sites, REV-ERBs response elements (RREs)/NGFI-B response elements (NBREs) (designated as R/N sites), which are recognized by REV-ERBα and NURR1 in the promoter region of the mouse TH gene [13]. However, in the hypoDAergic state of a PD model, the association between emotional disorders and disturbances of the molecular clock remains to be elucidated.

In the present study, we selected the unilateral intrastriatal 6-hydroxydopamine (6-OHDA) mouse with mild motor dysfunction as a PD model [16, 17], considering that non-motor symptoms in PD appear across all disease stages. We assessed the circadian time dependency of depression- and anxiety-like behaviors in 6-OHDA-injected mice. Since the circadian nuclear receptor REV-ERBα in the ventral tegmental area (VTA) is important for daily mood-related behavior through directly regulating DAergic system [13], we assessed whether the pharmacological administration of a REV-ERBα antagonist (SR8278) [18] into the VTA could change the behavioral symptoms of a PD mouse model in a time-dependent manner. We demonstrate the mechanism of action of SR8278, focusing on crosstalk and genome-wide motif enrichment of REV-ERBα and NURR1.

## Methods

### Animals and Surgery

Male C57BL/6 J wild type (WT) mice and dopamine transporter (DAT)-cre knock-in mice (Jackson Laboratory, [B6.SJL-*Slc6a3*^*tm1.1(cre) Bkmn*^/J, Strain #006660]) were obtained at an age of 8–10 weeks. The mice were housed using a 12-h/12-h light–dark (LD) photoperiod (light on at 10:00 a.m.) at a constant temperature (22–23 °C). Food and water were provided ad libitum. All procedures were approved by our institution’s Animal Care and Use Committee at DGIST (Daegu Gyeongbuk Institute of Science and Technology) (DGIST-IACUC-20102305–000). Unilateral striatal 6-OHDA-lesioned mice were used as a PD animal model as previously described [17] with minor modifications. Animals were deeply anesthetized using pentobarbital sodium (50 mg/kg, intraperitoneal injection) and placed in a stereotaxic device (Stoelting). A single injection of 6-OHDA (15 µg in 1.0 µL of 0.9% saline containing 0.02% ascorbate) or vehicle (1.0 µL of 0.9% saline containing 0.02% ascorbate) was administrated into the left dorsal striatum (anterior–posterior (AP): + 0.6 mm, medial–lateral (ML): −2.0 mm, dorsal–ventral (DV): −3.5 mm form the bregma) [19] using a 26-guage micro-syringe at a rate of 0.5 µL/min. After ~ 5 weeks post injection, the mice were kept in DD condition for 3–4 days where they acclimatized to DD condition (to eliminate light influence), starting at the time at which lights were turned off. Next, several behavioral tests were performed under a dim red light (DD condition). The experimental time windows were limited (3 h at dawn and dusk every day) and each test required approximately 2–3 days. After that, mice were sacrificed at the indicated time points under a dim, red light by cervical dislocation.

### Local Injection of SR8278

The local injection of SR8278 into the midbrain towards the VTA was performed 3 h before each behavioral test under a dim red light. We followed the time-regiment, for handling animal care and slow microinjection of SR8278 to the VTA 3 h before behavioral tests, as shown previously [13]. SR8278 (Tocris Bioscience) was dissolved in ethanol to a concentration of 50 µg/µL. SR8278 (20 µg/mouse) or the corresponding vehicle (ethanol) was directly microinfused into the VTA using a 33-gauge injector cannula (model C315I; PlasticOne) attached to a 10-µL Hamilton syringe at a rate of 0.1 µL/min. For the microinfusion, mice were anesthetized with sodium pentobarbital (50 mg/kg, i.p.), mounted on a stereotaxic apparatus (Stoelting), and unilaterally implanted with a 26-guage stainless steel cannula (model C315G, PlasticOne) into the midbrain towards VTA (AP −3.2 mm, ML −0.5 mm, DV −3.5 mm). A 32-gauge dummy cannula was inserted into each guide cannula to prevent clogging. Once the jewelry screws were implanted in the skull as anchors, the whole assembly was affixed to the skull with resin cement.

### Behavioral Studies

All behavioral tests were carried out blinded to the experimental group at the indicated time of day (CT22-01 vs. CT10-13) under a dim red light as previously described [13].

#### Tail-Suspension Test (TST)

Mice were suspended by the tail with an adhesive tape in a white box (36.5 × 30.5 × 30.5 cm) and left in this position for 6 min. The behavior of each mouse was video-recorded. The immobility time was defined as the time of agitation and a cessation in the escape attempts was considered an index of depression. Immobility was scored as the percentage of time spent immobile during the last 4 min of the test.

#### Forced Swim Test (FST)

To quantify depression-like behavior, mice were placed in a glass cylinder (10 cm diameter) filled with water (21–25 °C) at a depth of 15 cm. The testing period lasted for 6 min. Immobility was defined as motionless floating or the absence of limb movement in water. Immobility was scored as the percentage of the time spent immobile during the last 4 min of the test.

#### Elevated Plus Maze (EPM) Test

The EPM was performed as previously described [13] with minor modifications. The EPM apparatus was composed of two open (5 × 30 cm) and two enclosed (5 × 30 cm) arms located 50 cm above the ground. At the beginning of the trials, mice were individually placed in the center of the apparatus facing an open arm and recorded by an overhead video camera connected to a computer with tracking software (Ethovision 13, Noldus) for 10 min. The duration of arm entries and total distance were measured using Ethovision. The frequency of the arm entries and the number of center crossings were counted. An entry was defined as the movement of all four paws into an arm. The tendency to avoid the open arms is considered an index of anxiety-like behaviors.

#### Cylinder Test

Locomotor asymmetry was examined based on forelimb-use asymmetry on landing during the cylinder test [20]. Mice were placed individually in a glass cylinder (diameter: 11 cm, height: 30 cm) for 5 min, and the number of forepaw landing contacts was counted. Forelimb preference was scored as [*R* / (*L* + *R*)] × 100, where *L* is the number of left forepaw contacts and *R* is the number of right forepaw contacts.

#### Rotarod Test

Motor coordination was evaluated by measuring the time spent on an accelerating rotarod (B.S. Technolab Inc.) [21]. Before the tests, mice were trained for 5 min on the rotarod at 4 rpm and then allowed to rest for ≥ 60 min. After the training and rest, mice were tested in three trials with intervals of ≥ 30 min. During each trial, the rod accelerated from 4 to 40 rpm over a 5-min period, and the time to falling was recorded. The average times from the three trials were calculated for the analyses.

### Circadian Rhythm of Locomotor Activity and Body Temperature

The E-Mitter (Mini Mitter) is a battery-free and implantable telemetry system that allows for the real-time monitoring of temperature and motor activity. Mice were anesthetized with isoflurane and an E-mitter was inserted beneath the skin on the back of the mice. Mice were caged in a plexiglas box (35 × 18.5 × 14 cm), and body temperature and locomotor activity were recorded at 6-min intervals [22]. In this set of experiment, to monitor the locomotor activity and body temperature, mice were kept under a standardized light condition during 2 weeks for entraining and recovery period, as described previously [22]. The period, amplitude, and robustness of rhythms were determined by a cosinor fitting analysis of oscillation profiles during the entrainment period (L:D = 12 h:12 h), while the free-running period (FRP) was assessed by oscillation profiles under constant darkness condition.

### Virus Preparation

The recombinant adeno-associated viral (AAV) vector expressing cre-dependent mGFP and synaptophysin-mRuby (AAV1-hSyn-FLEx-mGFP-2A-Synaptophysin-mRuby, 1.38 × 10^13^ genomic copies/mL) was generated in-house as previously described [23] with modifications. The AAV plasmid, pAAV-hSyn-FLEx-mGFP-2A-Synaptophysin-mRuby, was a gift from Liqun Luo (Addgene plasmid # 71,760) [24].

### Immunohistochemistry

Mice were anaesthetized with pentobarbital sodium (50 mg/kg, i.p.) and perfused transcardially with phosphate-buffered saline (PBS, PH 7.6) containing 4% paraformaldehyde, after which the brain was isolated. Immunohistochemistry was performed as previously described [25]. Following post-fixation in the same fixative overnight, brains were cryoprotected in 30% sucrose for 48 h at 4 °C. Frozen coronal sections were then cut at a thickness of 20 µm using a cryostat (Leica). Sections containing the substantia nigra pars compacta (SNpc) and the VTA were mounted and antigen retrieval was performed in sodium citrate buffer heated to 100 °C for 10 min. After several washes in PBS containing 0.1% Triton X-100, sections were incubated in blocking solution (0.3% Triton X-100, 10% normal goat serum in PBS) for 60 min. After washing with PBS containing 0.1% Triton X-100, sections were incubated overnight 4 °C with anti-TH (Abcam, ab112) antibody. After several washes, biotinylated secondary antibody was applied for 1 h. Avidin–biotin-peroxidase complex (Vectastain ABC kit; Vector Laboratories) and 3–3′-diaminobenzidine reactions were used to visualize labeled proteins. TH staining sections were observed with an Eclips 90i microscope (Nikon). The number of TH-positive neurons was assessed using the optical dissector method of stereological analysis. Using Stereo Investigator software (MicroBrightField), a fractionator probe was established for each section and six sections covering the SNpc and the VTA were examined. The border separating the SNpc and the VTA was delineated at a lower magnification. The number of TH-positive neurons in each counting frame was determined by focusing down through the section using a × 40 objective lens, and the total numbers of positive neurons in the SNpc and VTA were calculated using the Stereo Investigator software. Image acquisition and counting of TH-positive neurons were performed by the observer blinded to the experimental group.

### Western Blot Analysis

To quantify the protein expression of genes of interests, the mice were sacrificed at the indicated time of day, and the brain tissues were rapidly extracted, frozen in dry ice, and stored at −70 °C before use. A western blot analysis was performed as previously described [13] with modifications. Protein samples were resolved on sodium dodecyl sulfate (SDS)-polyacrylamide gels and transferred to PVDF membranes (Millipore) in a Bio-Rad Trans-Blot electrophoresis apparatus using Towbin’s buffer (25 mM Tris [pH 8.3], 192 mM glycine, and 20% methanol). Blots were blocked in Tris-buffered saline (TBS; 10 mM Tris [pH 7.6], 150 mM NaCl, and 2 mM MgCl_2_) containing 0.3% Tween-20 and 5% bovine serum albumin (BSA) and incubated with anti-TH (Sigma Aldrich, T2928) or anti-actin (Santa Cruz Biotechnologies, sc-47778) antibody at room temperature for 1 h. Blots were then washed four times with TBS containing 0.3% Tween-20. Antibody binding was subsequently assessed by incubation with secondary antibodies linked to horseradish peroxidase (Jackson ImmunoResearch Laboratories). Blots were again washed several times, and immunoreactive bands were visualized by exposure to X-ray film or using a ChemiDoc XRS system (Bio-Rad) after the application of enhanced chemiluminescent (ECL) reagents (Thermo Fisher Scientific).

### Fluorescent In Situ Hybridization (FISH)

Brains were dissected rapidly from mice at the indicated time. For preparing the cryosections, fresh tissues were placed and snap-frozen in isopentane precooled in dry ice. Frozen coronal sections were cut at a thickness of 14 µm using a cryostat. Sections containing ipsilateral VTA regions were mounted onto Superfrost Plus Microscope Slides (Fisher Scientific). The sections were fixed in 4% paraformaldehyde for 10 min, dehydrated in increasing concentrations of ethanol for 5 min, and finally air-dried. Tissues were then pretreated for protease digestion for 10 min at room temperature. Mm-*Th*-C3 (Cat No. 317621-C3), Mm-*Nr4a2*-C1 (Cat No. 423351), and Mm-*Nr1d1*-C2 (custom probe) targeting 301–1404 of NM_145434.4 (Advanced Cell Diagnostics) were used to detect mRNA for TH, Rev-erbα, and Nurr1, respectively. Probe hybridization and amplification were performed at 40 °C using HybEZ hybridization oven (Advanced Cell Diagnostics, Hayward, CA). The sections were hybridized with the labeled probe mixture at 40 °C for 2 h per slide. The amplification and detection of the signal were performed using a commercial kit (Advanced Cell Diagnostics). All images were acquired using a Zeiss LSM 800 laser scanning confocal microscope (Carl Zeiss, Oberkochen, Germany) with the following settings: images were acquired using a Plan-Apochromat 20 × objective (*NA* = 0.8), with bidirectional scanning (a pinhole of 1 Airy unit). The scan range was 2048 × 2048 pixels, 16 bits/pixel. Fluorescent probes were excited using 405, 488, 561, and 647 nm lasers for the detection of DAPI, Rev-erbα mRNA, Nurr1 mRNA, and TH mRNA, respectively. The emitted fluorescence was collected using the GaAsP PMTs (selectively collect fluorescence at 465, 517, 576, and 670 nm for DAPI, Rev-erbα mRNA, Nurr1 mRNA, and TH mRNA, respectively). Images were acquired by the observer blinded to the experimental group. ImageJ program (NIH) was used to analyze the images. mRNA particles were assessed in the images from two sections covering the VTA in each animal. The number of *Rev-erbα* and *Nurr1* mRNA spots in the TH + neurons by defining the region of interest (ROI) in an image was counted using Analyze Particles from ImageJ software. The number of ROIs per each section varied from 3 − 26, and 11 ROIs were analyzed on average per each section. The absolute number of transcripts in single TH + cells in an image was divided by the number of TH + neurons.

### Chromatin Immunoprecipitation (ChIP) Assay

Brain tissues containing ipsilateral SN and VTA were isolated from the mouse brain after the sacrifice at the indicated time. ChIP assays were performed using a commercial assay kit (cell signaling). Chromatin prepared from midbrain tissues containing SN and VTA regions from two animals for each group was sheared and pre-cleared. Next, chromatin was divided and immunoprecipitated overnight at 4 °C by rotation with anti-REV-ERBα (cell signaling, #13,418) and anti-NURR1 (Santacruz, sc-376984) antibody. Immune complexes were collected by incubation with protein G magnetic beads (Cell Signaling). qPCR was performed using a TaqMan**™** Universal PCR Master Mix (Applied Biosystems). The primer sets used for the Taqman qPCR assay were as previously described [13]. TH up, 5′-AGA GCA GGC AGT GCA GAG TA-3′; TH dn, 5′-TTC CTG TTC CAG GAG TCT GA-3′; TH probe, 5′FAM-CCA GCT TGG CGC AGC ATC TC-3′BHQ1 for REV-ERBα; TH up, 5′-GCC GAG ACA AGA GAA CGA AT-3′; TH dn, 5′-CAG CAA CAC GAA TGT GGA TT-3′; TH probe, 5′FAM-CCT TTC GGC AAA TGT CTT ACA CTG ACA-3′BHQ1 for NURR1.

### Sample Preparation for ATAC Sequencing

Adult mice were entrained at 12-h/12-h light–dark (LD) photoperiod. 6-OHDA-lesioned and its control mice were prepared as described above. The VTA regions were dissected into 1-mm thickness using brain matrix then sampled using 1-mm micropunch in pre-chilled Hanks’ Balanced Salt Solution (HBSS; Gibco). VTA samples were collected from five mice per group at ZT00 and at ZT12. The VTA tissues were washed with 1 mL of fresh neurobasal media (Gibco) supplemented with B27 (Gibco) and 25 mM HEPES (Gibco). Then, the samples were incubated in dissociation buffer containing 1 mg/mL papain (Worthington Biochemical Corporation), B27, and 25 mM HEPES diluted in neurobasal media for 30 min at 37 °C with gentle agitation. After incubation, gentle trituration was performed by pipetting with 1-mL pipettes for 25 times. The procedures for transposition reaction, purification, and library preparations were performed as described [26]. Libraries were quantified by real-time qPCR and sequenced on a single lane of Hi-seq 2500 (Illumina) using 100 bp paired-end reads (Macrogen).

### Data Processing for ATAC Sequencing

The sequencing files were first checked for quality control using FastQC (http://www.bioinformatics.babraham.ac.uk/projects/fastqc). Trimmomatic [27] was used to remove the adapter sequences before mapping with Bowtie2 with mus musculus reference genome (mm10), parameters “–very sensitive –dovetail” [28]. And then we sorted the unmapped and mapped reads and mapping quality below 10 was eliminated with SAMtools version 1.12 [29]. To remove the PCR duplication during library preparation, sorted reads were undergone with Picard deduplication (http://broadinstitute.github.io/picard/). Before the further analysis, the final reads were checked with FastQC again and proceeded when the quality control is acceptable.

ATAC-seq peaks were called using HOMER (http://homer.ucsd.edu/homer/ngs/index.html) with FDR 0.05 as thresholds. Called peaks were visualized with the UCSC genome browser. For annotating and motif analysis from the called peaks, their genomic regions, and REV-ERBα and NURR1 motifs from JASPAR database MA1531.1 and MA0160.1, respectively, were processed with annotatePeaks using HOMER. For the post-alignment quality control, ATACseqQC was perfumed to check the fragment sizes of ATAC-seq libraries [30], and chromatin accessibility from transcription start sites was visualized with deepTools version 3.5.1 [31].

### Statistical Analysis

All data were reported as mean ± SEM. Inter-group differences were evaluated using an unpaired two-tailed Student *t* test, a one-way analysis of variance (ANOVA) followed by a post-hoc comparison using Bonferroni test, a two-way ANOVA, and a two-way repeated measured ANOVA followed by a post-hoc comparison using a Newman-keuls for behavior test and a Fisher’s LSD for molecular analysis. All analyses were performed using GraphPad Prism (version 7.0; GraphPad Software), and differences were considered statistically significant at a *p*-value of < 0.05.

## Results

### Pharmacological Inhibition of REV-ERBα Activity with SR8278 Restored Circadian Mood-Related Behaviors in PD Mouse Models

**Fig. 1:**
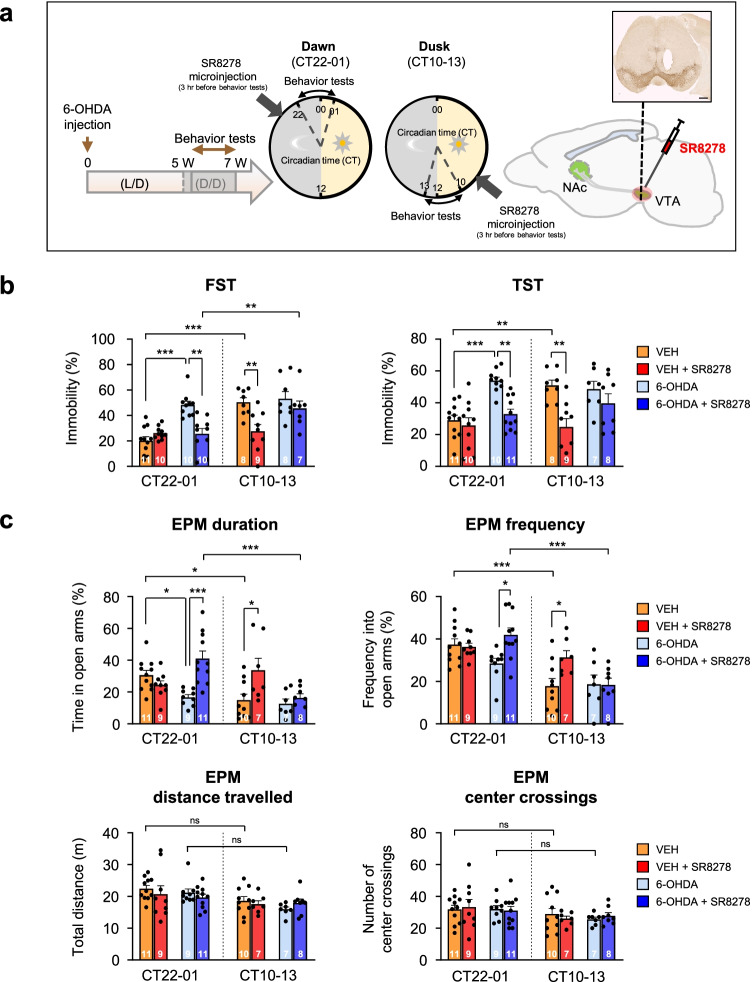
SR8278 treatment recovered mood-related behavioral deficits shown in 6-OHDA-lesioned mice. (**a**) Experimental scheme of behavior tests. A single injection of 6-hydroxydopamine (6-OHDA) was administrated into the left dorsal striatum. After 5 weeks post injection, mood-related behavioral tests were carried out at the indicated time of day (circadian time [CT] 22–01 vs. CT10-13). The local injection of SR8278 (20 μg/mouse) into the midbrain was performed 3 h before each behavioral test. The image was used to confirm cannula placement in the VTA by TH staining to show DA neurons in the SN and VTA (inset). Scale bar = 500 μm. (**b**) Changes in depression-like behaviors after SR8278 microinfusion into the midbrain of VEH- and 6-OHDA-treated mice by a forced swim test (FST) and tail suspension test (TST) (FST: two-way ANOVA, *p* < 0.0001 for injection group, *p* < 0.0001 for time, *p* = 0.0021 for interaction) (TST: two-way ANOVA, *p* < 0.0001 for injection group, *p* = 0.0624 for time, *p* = 0.0105 for interaction). (**c**) Alternation of anxiety-like behaviors after SR8278 administration of VEH- and 6-OHDA-treated mice by an elevated plus maze test (EPM) (duration: two-way ANOVA, *p* = 0.0021 for injection group, *p* = 0.0025 for time, *p* = 0.0005 for interaction) (frequency: two-way ANOVA, *p* = 0.0240 for injection group, *p* < 0.0001 for time, *p* = 0.0182 for interaction) (distance traveled: two-way ANOVA, *p* = 0.5047 for injection group, *p* = 0.0013 for time, *p* = 0.6996 for interaction) (center crossings: two-way ANOVA, *p* = 0.9616 for injection group; *p* = 0.0245 for time; *p* = 0.8537 for interaction). Data are presented as mean ± SEM. Sample sizes (animals) are indicated by the numbers inside bars. Newman-keuls corrected post-hoc comparisons are indicated by *p < 0.05, **p < 0.01, ***p < 0.001 and *ns*, non-significant

To assess whether daily variations in mood-related behaviors were altered in the striatal 6-OHDA-lesioned PD mouse model, we observed the emotional behavioral phenotypes in vehicle (VEH)-injected or 6-OHDA-lesioned mice, at subjective dawn [circadian time (CT) 22–01] and dusk (CT10-13) at 5 weeks after the 6-OHDA lesion (Fig. 1a). To demonstrate that regulation of REV-ERB activity could restore changes in mood-related behaviors in 6-OHDA-lesioned mice, we observed the effect of the REV-ERB antagonist SR8278 on affective behaviors in 6-OHDA-lesioned mice by local administration of SR8278 into the VTA at 3 h before behavioral tests. We tested the dose–effect of SR8278 on depression-like behavior in WT mice; 20 μg/mouse of SR8278 was effective in suppressing immobility at CT10-13 by FST (Supplementary Fig. 1).

In the despair-based FST and TST, VEH-injected control mice showed time-of-day variation in depression-like behaviors (Fig. 1b). However, 6-OHDA-lesioned mice exhibited a significant increase in immobility time only at CT22-01, with the disappearance of daily variations compared to the control groups. Interestingly, SR8278 microinjection into the VTA significantly suppressed the immobility times in the FST and TST, which were increased by a 6-OHDA lesion at CT22-01, showing an antidepressant effect in 6-OHDA-lesioned mice, although SR8278 did not affect the immobility time in VEH-treated mice (Fig. 1b). Unlike the effects of SR8278 at CT22-01, the SR8278 microinfusion significantly decreased the immobility time and exerted antidepressant effects in VEH-treated mice, but not in 6-OHDA-injected mice at CT10-13. SR8278 treatment rescued depression-like behaviors in 6-OHDA lesioned mice only at CT22-01, recovering the circadian pattern of FST and TST scores resembling those of VEH-injected control mice.

The EPM test revealed that 6-OHDA-injected mice showed more anxiety-like phenotypes only at CT22-01, with a shorter duration, leading to the elimination of circadian patterns of behavior shown in VEH-treated mice (Fig. 1c, upper panels). In the EPM test, the SR8278 microinfusion increased the duration and frequency in the open arms in 6-OHDA-lesioned mice only at CT22-01, exerting anxiolytic effects; however, this was not observed in VEH-treated mice. Anxiety-like behaviors were reduced in VEH-injected mice only at CT10-13 by SR8278 microinjection. SR8278 recovered the rhythm of anxiety-like behaviors in 6-OHDA-lesioned mice, similar to that observed in VEH-treated mice. There were no significant differences in the distance traveled and center crossings in the EPM with 6-OHDA lesion and SR8278 treatment, suggesting that anxiety responses in EPM are not attributable to changes in general activity (Fig. 1c, bottom panel). Our results revealed that SR8278 treatment recovered emotion-related disorders in a circadian time-dependent manner in 6-OHDA-lesioned mice, exerting antidepressant and anxiolytic effects, especially at dawn.

To confirm that 6-OHDA lesions induced PD-like motor deficits, we performed cylinder and rotarod tests (Supplementary Fig. 2a, b). The cylinder and rotarod tests revealed that 6-OHDA-injected mice exhibited significant motor asymmetry and decreased motor coordination performance regardless of the time of day. Furthermore, we assessed the circadian rhythmicity of active-rest cycles and body temperature in both LD and constant dark (DD) conditions (Supplementary Fig. 3a–c). The variation between light and dark periods in total activity and body temperature in the LD condition decreased at 2 weeks following 6-OHDA microinjection. Furthermore, 6-OHDA-lesioned mice showed a longer period, lower amplitude, and lower robustness of rhythm in locomotor activity and body temperature under DD conditions, exhibiting a disturbance of endogenous rhythm (Supplementary Fig. 3a, d–e).

### SR8278 Microinjection Alters Remaining DAergic Neuron-Specific Transcription Levels of Rev-erbα and Nurr1 in the VTA

To prove that 6-OHDA-lesioned mice show significant PD pathologies in DAergic neurons, we analyzed TH expression in both SNpc and VTA (Supplementary Fig. 4). Immunohistochemistry revealed that control mice exhibited a temporal variation with a greater number of TH + neurons at CT00 than at CT12 in both the SNpc and VTA (Supplementary Fig. 4b). The ipsilateral 6-OHDA-lesioned mice exhibited a significant decrease in the number of TH + neurons in the SNpc and VTA, regardless of the time of day, compared to the contralateral region or the VEH-injected control animals. Furthermore, 6-OHDA injection led to the disappearance of any temporal variation in the number of TH + neurons on the ipsilateral side. The degree of TH + neuronal loss on the ipsilateral side of the 6-OHDA-lesion mice was more severe in the SNpc than in the VTA, which is consistent with previous findings [16, 32]. Western blot analysis also supported these findings (Supplementary Fig. 4c, d). To confirm the DA neuronal loss in 6-OHDA lesioned mice with TH-independent marker, we microinjected AAV1-hSyn-FLEx-mGFP-2A-Synaptophysin-mRuby into the SNpc/VTA of DAT-cre knock-in mice to visualize the changes in the number of mGFP-tagged DAergic neurons and mRuby-tagged DA neuron-specific synaptophysin (Supplementary Fig. 5a). DA neuronal- and its synaptic loss were observed in the SNpc and VTA of striatal 6-OHDA lesioned mice (Supplementary Fig. 5b). The effects of 6-OHDA on DAergic neurons were more severe in the SNpc than in the VTA, which is consistent with our immunohistochemical and western blotting data (Supplementary Fig. 4). To assess whether 6-OHDA lesions altered the DAergic system and circadian clock, we assessed the circadian mRNA expression of DA-related genes and circadian clock genes in the midbrain of 6-OHDA-lesioned mice (Supplementary Fig. 6). *TH* and *Bmal1* mRNA levels were elevated at dawn, which differed from the circadian oscillation of *Per2 m*RNA levels in the control. The expression levels of *Nurr1* mRNA were constantly expressed, regardless of the time of day. *TH* and *Nurr1* mRNA expression were reduced by 6-OHDA lesions (*p* < 0.0001 for *TH*, *p* < 0.0001 for *Nurr1*), whereas transcriptional levels of circadian clock genes, *Bmal1* and *Per2*, were largely unaffected by 6-OHDA, showing their circadian patterns (*p* = 0.0002 for *Bmal1*, *p* = 0.0156 for *Per2*).

**Fig. 2:**
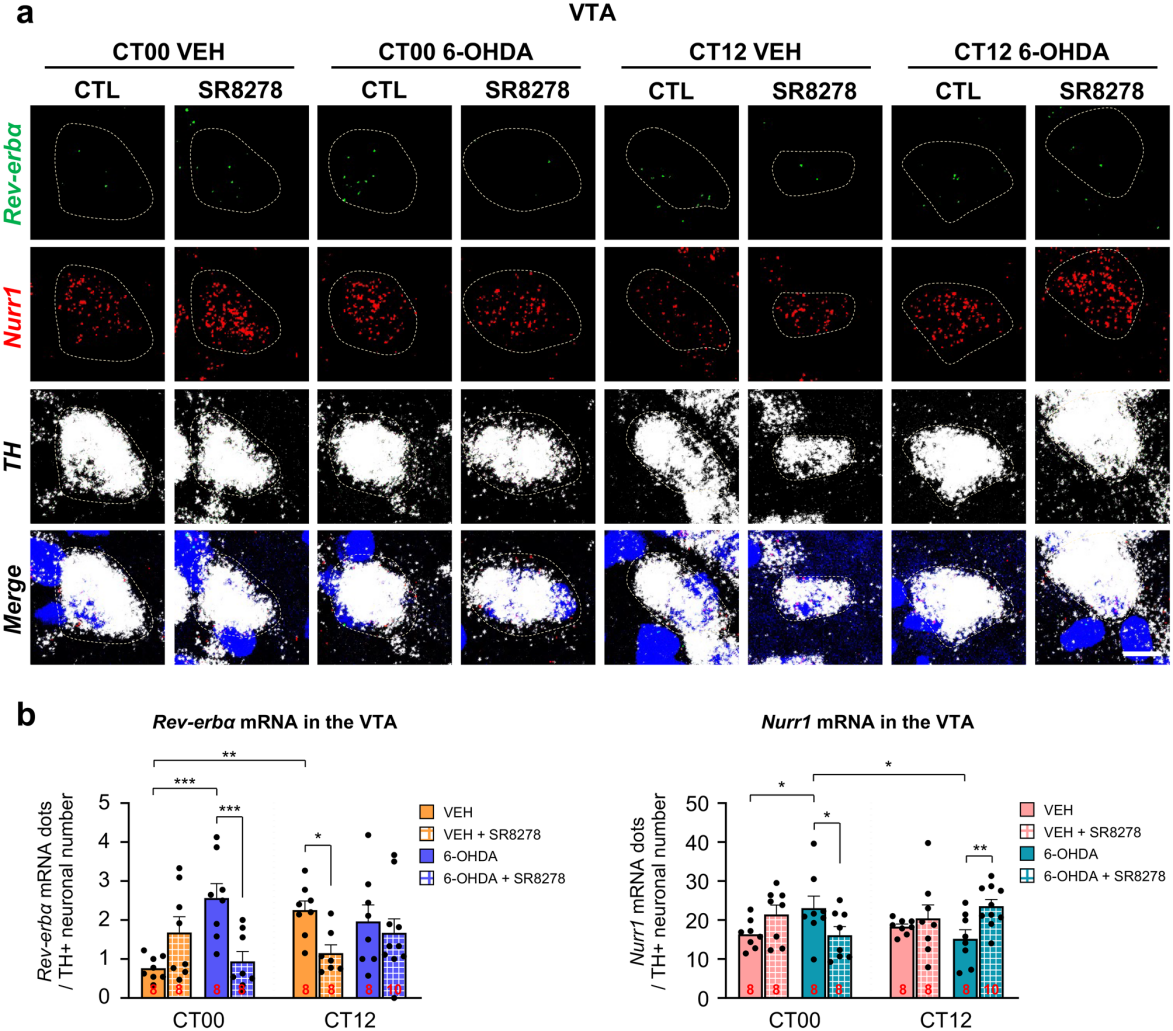
SR8278 microinjection altered transcription levels of DAergic neuron-specific *Rev-erbα* and *Nurr1* in the VTA. (**a**) Representative FISH images of VTA with *Rev-erbα*, *Nurr1*, and *TH* staining. Scale bar = 10 µm. (**b**) *Rev-erbα* and *Nurr1* mRNA expression in the VTA was represented by mRNA particles in the TH + neurons divided by the counts of TH + neurons. mRNA particles in TH + neurons were analyzed by defining the region of interest (ROI). mRNA particles were assessed in the images from two sections, covering the VTA in each animal. (*Rev-erbα*: two-way ANOVA, *p* = 0.0172 for injected group, *p* = 0.2354 for time, *p* = 0.0042 for drug-by-time interaction) (*Nurr1*: two-way ANOVA, *p* = 0.4650 for injected group, *p* = 0.9380 for time, *p* = 0.0115 for drug-by-time interaction). The data were presented as mean ± SEM. Sample sizes (brain sections) are indicated by the numbers inside bars. Fisher’s LSD post-hoc comparisons are indicated by *p < 0.05, **p < 0.01, and ***p < 0.001

To study the mechanisms of action underlying SR8278-induced behavioral recovery in the 6-OHDA-lesioned mice, we measured the remaining DAergic-specific transcription levels of *Rev-erbα* and *Nurr1*, which are the upstream nuclear receptors of *TH*, in the VTA after SR8278 microinjection, as assessed by FISH analysis (Fig. 2a). *Rev-erbα* mRNA expression in the TH + neurons of the VTA was significantly affected by 6-OHDA or SR8278 treatment (*p* = 0.0172) and drug-by-time interaction (*p* = 0.0042) (Fig. 2a, b, left panel). In the control group, *Rev-erbα* mRNA levels were significantly elevated at CT12 compared to CT00. However, 6-OHDA lesions increased *Rev-erbα* transcript levels only at CT00 without altering the mRNA levels at CT12, resulting in the loss of daily variation in *Rev-erbα* transcript levels compared to VEH-treated mice. SR8278 treatment significantly reduced *Rev-erbα* mRNA expression in VTA DAergic neurons in 6-OHDA-lesioned mice at CT00, but not at CT12, thereby restoring rhythmic transcription of *Rev-erbα*. *Rev-erbα* mRNA expression was downregulated by SR8278 microinjection in the VTA DAergic neurons of VEH-treated mice at CT12, but not at CT00, showing time-dependent action of SR8278. *Nurr1* transcription levels in the remaining DAergic-specific neurons of the VTA were also affected by drug-by-time interaction (*p* = 0.0115) (Fig. 2a, b). VEH-treated mice showed constant transcription levels of *Nurr1*, regardless of the time of day. 6-OHDA lesions slightly increased DAergic-specific *Nurr1* mRNA levels at CT00 without an alternation at CT12, thereby disturbing the consistency of *Nurr1* transcription. However, SR8278 treatment lowered *Nurr1* mRNA levels in 6-OHDA-lesioned mice at CT00 and upregulated its level at CT12, showing time-dependent action of SR8278. These data suggest that the mRNA levels of the remaining DAergic-specific *Rev-erbα* and *Nurr1* in the VTA were altered in a time-dependent manner by 6-OHDA lesions. Notably, SR8278 treatment restored the time-dependent transcription levels of *Rev-erbα* and *Nurr1* in 6-OHDA-lesioned mice at dawn, consistent with the behavioral recovery (see Fig. 1).

### SR8278 Microinjection Restores Antagonistic Crosstalk of REV-ERBα and NURR1 Binding Activity to TH Promoter and TH Protein Levels in VTA at Dawn

**Fig. 3:**
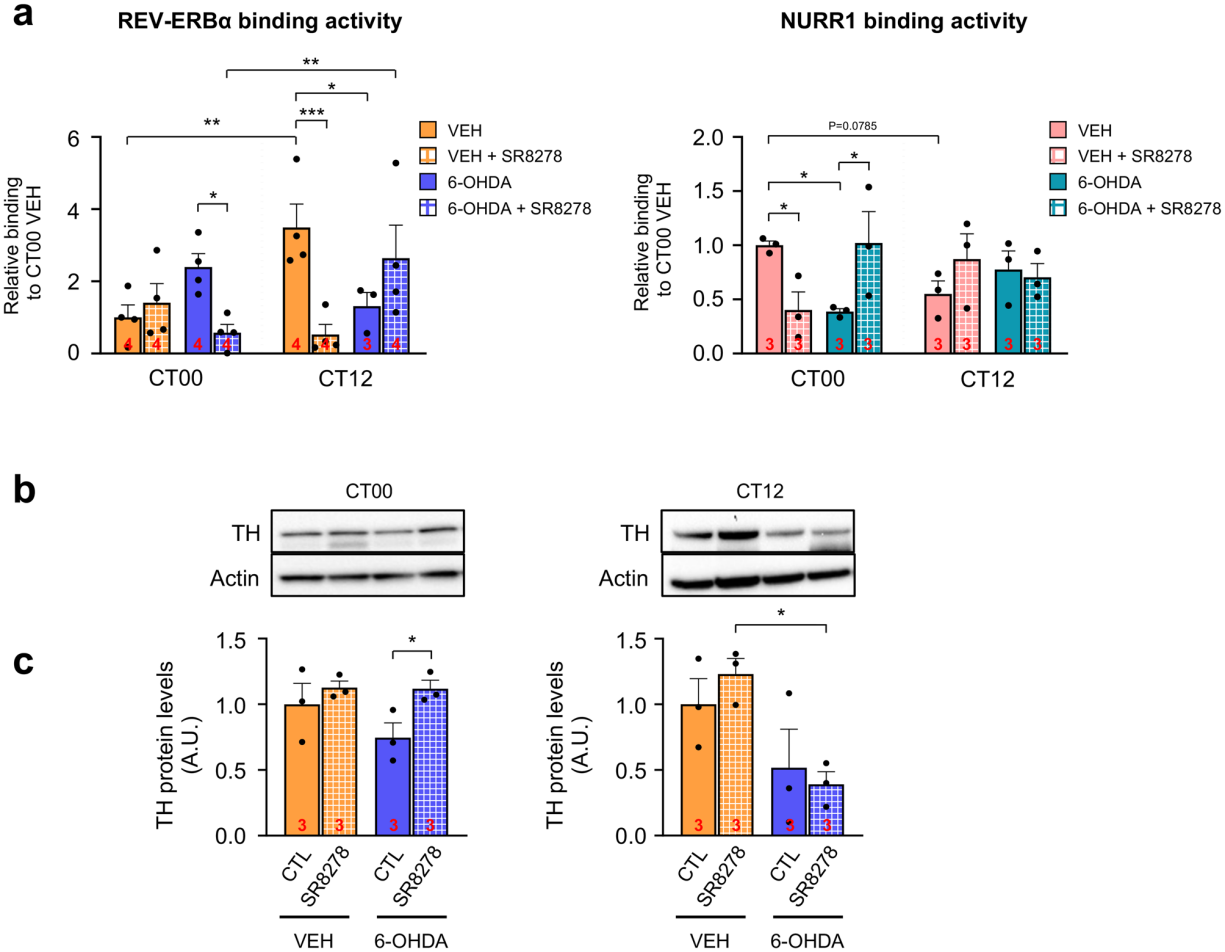
SR8278 restored binding activities of REV-ERBα and NURR1 and elevated TH expression at dawn. (**a**) REV-ERBα-bound *TH* promoter in midbrain lysates by ChIP-qPCR (left panel). NURR1-bound *TH* promoter in midbrain lysates by ChIP-qPCR (right panel). Chromatin prepared from midbrain tissues containing SN and VTA regions from two animals for each group. The binding activity was calculated as a percent of total chromatin used for immunoprecipitation (% input) and was normalized to VEH-treated control mice at CT00 (REV-ERBα: two-way ANOVA, *p* = 0.1166 for injected group, *p* = 0.0927 for time, *p* = 0.0023 for drug-by-time interaction) (NURR1: two-way ANOVA, *p* = 0.3594 for injected group, *p* = 0.8492 for time, *p* = 0.0284 for drug-by-time interaction). (**b**) Representative immunoblot images showing TH protein expression in the VTA derived from VEH- and 6-OHDA-lesioned mice at CT00 and CT12. Sample sizes (chromatin from two animals) are indicated by the numbers inside bars. (**c**) TH protein levels in VTA. After densitometric analysis, TH protein levels were normalized with actin levels (CT00: two-way ANOVA, *p* = 0.2508 for 6-OHDA-lesion, *p* = 0.0468 for SR8278 treatment, *p* = 0.2801 for interaction) (CT12: two-way ANOVA, *p* = 0.0088 for 6-OHDA-lesion, *p* = 0.7924 for SR8728 treatment, *p* = 0.3816 for interaction). Sample sizes (animals) are indicated by the numbers inside bars. The data were presented as mean ± SEM. Fisher’s LSD post-hoc comparisons are indicated by *p < 0.05, **p < 0.01, and ***p < 0.001

We performed a chromatin immunoprecipitation (ChIP) assay with VTA tissues to determine whether the competitive binding of REV-ERBα and NURR1 to R/N sites of the *TH* promoter would be altered by the 6-OHDA lesion and restored by SR8278 microinjection into the VTA. The REV-ERBα binding affinity to R/N sites was significantly affected by the time of day and drug injection of 6-OHDA and SR8278 (*p* = 0.0023) (Fig. 3a, left panel). In VEH-treated mice, REV-ERBα binding affinity was much higher at CT12 than at CT00, in accordance with *Rev-erbα* mRNA expression (Fig. 2b). In 6-OHDA-lesioned mice, the REV-ERBα binding activity to R/N sites was increased at CT00, but decreased at CT12, inducing a reversed pattern compared to the VEH-treated mice. Notably, SR8278 treatment rescued REV-ERBα binding affinity to R/N sites by reducing its affinity in 6-OHDA-lesioned mice only at CT00. On the other hand, SR8278 treatment tended to increase REV-ERBα binding affinity at CT12 in 6-OHDA-lesioned mice, showing the time-dependent action of SR8278. In VEH-treated mice, SR8278 local injection significantly reduced REV-ERBα binding activity to R/N sites only at CT12, but not at CT00. Because of the time-dependent effect of SR8278, it restored the rhythmic REV-ERBα binding affinity to R/N sites of *the TH* promoter in 6-OHDA-lesioned mice, similar to the control.

For NURR1 binding activity, the injection of 6-OHDA and SR8278 altered relative binding to its cis-elements (R/N sites) in a time-dependent manner (*p* = 0.0284), which is the opposite pattern observed in the REV-ERBα ChIP assay (Fig. 3a, right panel). NURR1 binding activity was higher at CT00 than at CT12 in the VEH-treated mice. The NURR1 binding affinity to R/N sites was significantly diminished in 6-OHDA-lesioned mice at CT00, but not at CT12. Notably, SR8278 treatment rescued NURR1 binding affinity to R/N sites by increasing its binding affinity in 6-OHDA-lesioned mice at CT00, while NURR1 binding affinity to R/N sites was not altered at CT12. NURR1 binding affinity to R/N sites was decreased at CT00 by SR8278 treatment in the VEH-treated mice, with a tendency to increase its binding affinity at CT12. NURR1 binding activity patterns were opposite to REV-ERBα binding activity, exhibiting antagonistic crosstalk between REV-ERBα and NURR1.

Because REV-ERBα and NURR1 bind to the R/N sites as nuclear receptors regulating TH expression, we observed changes in TH protein levels in the VTA by western blot analysis. TH protein expression in the VTA was significantly affected by SR8278 microinjection at CT00 (*p* = 0.0468), whereas TH protein expression of VTA was affected by 6-OHDA lesions at CT12 (*p* = 0.0088), but not by SR8278 treatment (*p* = 0.7924), showing time-dependent action of SR8278 (Fig. 3b, c). Reduced TH protein level of VTA by 6-OHDA lesion was restored to the levels in VEH-treated mice only at CT00, while SR8278 did not affect TH expression at CT12 in 6-OHDA-lesioned mice. These data indicate that the recovery of antagonistic crosstalk between REV-ERBα and NURR1 causes the elevation of TH expression in 6-OHDA-lesioned mice at dawn.

### SR8278 Treatment Induces Enrichments of REV-ERBα and NURR1 Binding Motifs at Dawn

To further investigate the underlying mechanisms of SR8278 in TH expression, we performed an assay for transposase-accessible chromatin using high-throughput sequencing (ATAC-seq) and motif-based analysis for R/N sites. This genome-wide mapping of the chromatin accessibility technique is useful for detecting chromatin regions with increased accessibility, annotating the peak called regions, and comparing the enriched motifs in different experimental groups.

**Fig. 4:**
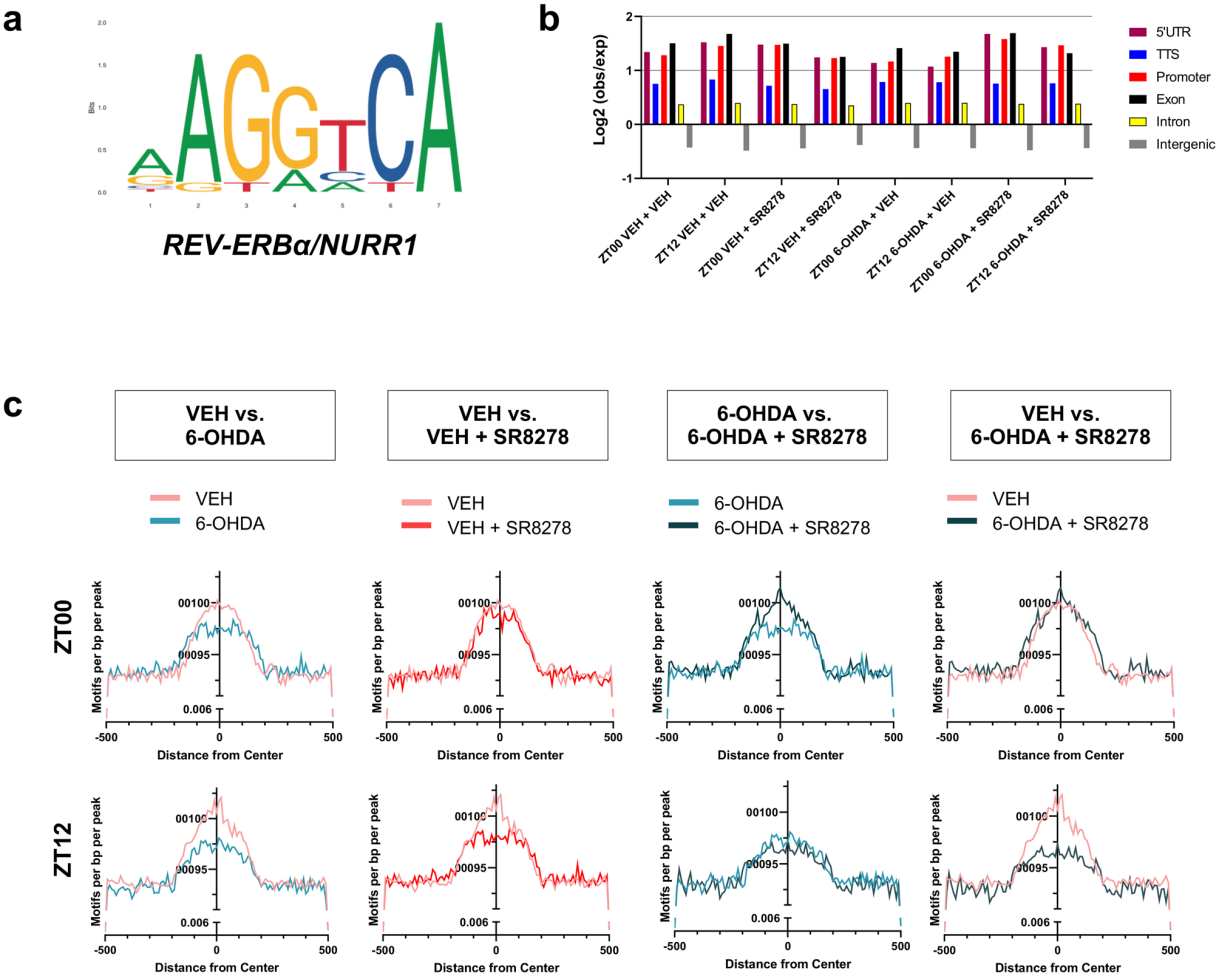
SR8278 microinjection to VTA altered enrichment of R/N motifs in 6-OHDA-lesioned mice only at dawn. (**a**) Sequence logos for REV-ERBα and NURR1 binding motifs are depicted [13]. (**b**) Log2 ratio (observed/expected) of ATAC-seq peaks was analyzed using HOMER. (**c**) Probability of R/N motifs were calculated using HOMER from called peaks and compared the motif enrichments at the indicated times for each experimental group. For each ATAC-seq data analysis, VTA regions were pooled from five individual mice (*n* = 5, pooled)

To include the effects of light cues and minimize the individual variations in the genomic architectures, we prepared the samples under the LD cycle, which is considered zeitgeber (the external cue), and pooled VTA samples from five animals for each group (*n* = 5). We observed an increase in TH gene expression at CT00 compared to CT12 (Fig. 3b, c), which agrees with the greater number of peaks and the chromatin accessibility of the TH genomic regions at zeitgeber time (ZT) 00 in VEH-treated groups compared to that at ZT12 (Supplementary Fig. 7). We acquired consistent results on TH expression under circadian (without light cues) and zeitgeber conditions. This allowed us to presume that the peak calling of ATAC-seq is adequate for further analysis. For additional quality controls for ATAC-seq results, the annotated genomic locations of the called peaks did not show clear differences in fold changes of transcription start sites (TSS) and promoter regions among experimental groups, and we presented fragment distributions for each ATAC-seq sample (Fig. 4b, Supplementary Fig. 8).

To understand the epigenetic regulation of SR8278 in 6-OHDA-lesioned mice, we characterized the differential motif analysis between experimental groups using the ATAC-seq approach. For this, we used the known motif for R/N with REV-ERBα and NURR1 binding cis-acting sites in the TH promoter, as previously described (Fig. 4a). We analyzed the R/N motifs using HOMER and compared the motif densities within 1 kb from the peaks between the indicated experimental groups, which were presented at a specific time point, at ZT00 or ZT12 (Fig. 4c). The occurrence of R/N motifs was clearly altered in the 6-OHDA and/or SR8278 treated groups. 6-OHDA-lesioned mice showed a notable reduction in R/N motifs at ZT00 and ZT12 compared to the VEH-treated mice (Fig. 4c, first panel). SR8278 treatment in 6-OHDA-lesioned mice increased the number of R/N motifs only at ZT00 (Fig. 4c, third panel). Moreover, the increase in the enrichment of R/N motifs by SR8278 in 6-OHDA-lesioned mice was almost similar to that in VEH-treated mice only at ZT00. The SR8278 treatment did not seem to be effective at ZT12 in R/N motif enrichment (Fig. 4c, fourth panel). For the control analysis, we also performed differential motif analysis with E-box, the most documented cis-element in circadian biology (Supplementary Fig. 9a). Enrichment of E-box motifs remained unaffected by 6-OHDA or SR8278 treatments and did not show time-dependent differences at ZT00 and ZT12 (Supplementary Fig. 9b, c). Taken together, SR8278 not only changed the binding activities of REV-ERBα and NURR1 in 6-OHDA-lesioned mice but also altered the R/N motifs at specific time points, at ZT00 (Figs. 3a and 4c).

## Discussion

**Fig. 5:**
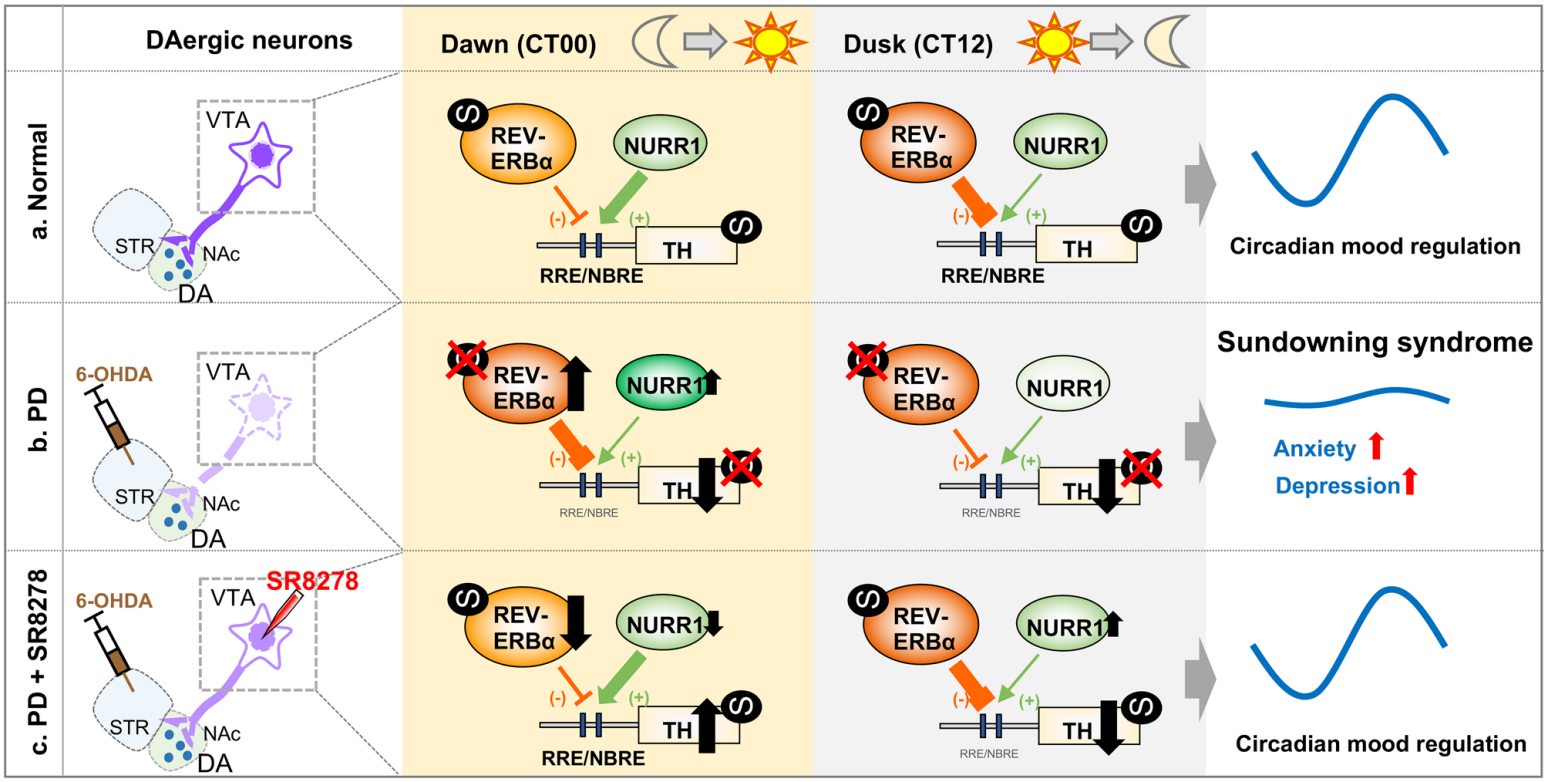
Mood regulation and crosstalk of REV-ERBα and NURR1 in PD and SR8278-treated PD model. (**a**) In the normal DAergic state, the circadian nuclear receptor REV-ERBα repressed the TH gene transcription via competition with NURR1, another nuclear receptor on the same cis-element (R/N sites), inducing rhythmic TH expression [13]. Circadian oscillation of TH expression in VTA neurons resulted in the daily variation of mood-like behaviors. (**b**) In the PD model induced by 6-OHDA-lesion, transcription level of *Rev-erbα* and *Nurr1* in the VTA DAergic neurons were altered at dawn, thereby inducing disappearance of rhythmic *Rev-erbα* transcription and disturbance of consistent Nurr1 transcription. Furthermore, 6-OHDA lesion induced atypical binding activity of REV-ERBα and NURR1 to R/N sites of *TH* promoter. REV-ERBα binding activity to R/N sites was increased at dawn but decreased at dusk. NURR1 binding activity was decreased at dawn without an alternation at dusk. In PD model, depression- and anxiety-like behaviors were exhibited at specific time, dawn, which time corresponds to dusk in diurnal human, characterizing the sundowning syndrome. (**c**) SR8278 microinjection completely restored rhythmic mood-related behaviors in 6-OHDA-lesioned mice at dawn, exhibiting the antidepressant and the anxiolytic effects in a time-dependent manner. Transcription levels of *Rev-erbα* and *Nurr1* in the VTA DAergic neurons and binding activities of REV-ERBα and NURR1 to R/N sites were recovered by SR8278 microinjection at dawn. TH protein levels of VTA were also elevated by SR8278 microinjection at dawn. Although the binding activities of REV-ERBα and NURR1 were restored at dusk, TH expression in VTA was not recovered by SR8278 at dusk. It is noteworthy that the competitive actions of REV-ERBα and NURR1 are essential in regulating the circadian *TH* gene expression and mood regulation

The present study clearly demonstrated that a unilateral intrastriatal 6-OHDA-injected mouse model of PD exhibits an increase in depression- and anxiety-like behaviors in a circadian time-dependent manner, specifically at dawn (see Fig. 5). Interestingly, the local microinjection of SR8278, a REV-ERBα antagonist, into the VTA had antidepressant and anxiolytic effects only at dawn in the 6-OHDA-lesioned mice, restoring the rhythmic mood-related behavioral patterns. 6-OHDA lesions altered the transcription levels of *Rev-erbα* and *Nurr1* in the remaining DAergic neurons of the VTA and atypical antagonistic crosstalk between two nuclear receptors, REV-ERBα and Nurr1, in a time-dependent manner. This is concomitant with arrhythmic oscillations of TH expression in the hypoDAergic state. SR8278 restored DAergic-specific transcription levels of *Rev-erbα* and *Nurr1*, and REV-ERBα and NURR1 binding affinities to R/N sites in 6-OHDA-lesioned mice at dawn, thereby increasing TH expression in the VTA region of 6-OHDA-lesioned mice at dawn. Similarly, SR8278 also altered the chromatin accessibility of the TH genome and R/N motif enrichment patterns in 6-OHDA-lesioned mice compared to the control groups, especially at dawn. These results suggest that REV-ERBα may be a novel molecular target and its antagonism (SR8278) may be a valuable drug candidate for the treatment of PD-related circadian behavioral disturbances, namely sundowning syndrome, thereby providing insights into circadian rhythm-based therapeutic interventions.

In sundowning syndrome, circadian patterns of behavioral disturbances are worse in the late afternoon/evening among the elderly and patients with dementia and neurodegenerative disorders over 60 years of age [33]. Although previous studies have revealed that aged and transgenic Alzheimer’s disease mouse models represent certain aspects of sundowning syndrome, such as increased locomotor activity and anxiety-like behaviors in the afternoon [34], the explanation for sundowning syndrome was inadequate because of the lack of an animal model and neurobiological mechanism studies associated with circadian rhythms. 6-OHDA-lesioned mice exhibit disturbances in their circadian patterns of mood-related behaviors with increased depression- and anxiety-like phenotypes only at dawn. These phenotypes are observed with sundowning syndrome in humans, because the period CT22-01 in nocturnal mice corresponds to the late afternoon/evening hours for humans, with the cessation of daily activity [34]. The endogenous circadian rhythm was disturbed in 6-OHDA-lesioned mice, which is consistent with a previous study [35, 36]. The present study demonstrated the pathophysiological molecular links between midbrain DAergic neurons and the circadian timing system, shedding light for the first time on the causal relationship between sundowning syndrome and a hypoDAergic state in a PD animal model. These results facilitate our understanding of the etiology of sundowning syndrome from a chronobiological aspect. Although animal research is needed to elucidate the mechanisms of the sundowning syndrome in PD, there are several limitations with application to clinical studies. Although the timing of sundowning syndrome is at the onset of an inactive period in both humans and mice, the disruptive behaviors of sundowning syndrome are expressed in the late afternoon and evening in humans, versus at dawn in mice, which are nocturnal species. Furthermore, sundowning syndrome is a complex behavioral disorder, including agitation, delusion, and hallucination [6]. Although we focused on depression- and anxiety-like disorders in PD mouse models, it is difficult to exactly replicate human complex psychiatric disorders in a mouse model.

The mood-related behavioral patterns shown in both 6-OHDA- and MPTP-lesioned mice were consistent with the comorbidity of anxiety and depression in patients with PD [37]. In toxin-based animal models of PD, controversial findings regarding changes in mood-related behaviors have been previously reported. For instance, several studies have reported increased depressive and anxiety-like behaviors in a 6-OHDA-lesioned animal model [38–40], consistent with our data. However, Branchi et al. [41] reported that bilateral injection of 6-OHDA into the dorsal striatum did not change the immobility time in the FST and rather decreased anxiety-like behaviors in the EPM. The MPTP lesion did not affect anxiety-related behaviors in the EPM [42, 43]. Explanations for these discrepancies may be the lack of circadian time-dependent measurements of mood-related behaviors, the dependence on toxin-lesion in discrete regions, and the neural circuits involved in the central DAergic system. Drui et al. reported that DAergic neuronal loss by the 6-OHDA lesion within the SNpc, but not within the VTA, may mediate affective disorders [39]. Although 6-OHDA induced severe neuronal loss in the SNpc, alongside the partial VTA neuronal loss in our study, in-depth studies are required to determine which discrete DAergic pathways mediate emotional instability in PD. VTA DAergic-specific *Rev-erbα* mRNA levels and its binding affinity to the TH promoter in the midbrain were dramatically upregulated at dawn in 6-OHDA-injected mice. We speculated that the increase in *Rev-erbα* mRNA may be associated with oxidative stress, which is considered a key trigger in the pathogenesis of PD [16]. It is well known that the circadian clock reciprocally interacts with redox homeostasis [44, 45], and oxidative stress increases *Rev-erbα* mRNA expression after hydrogen peroxidase treatment in mouse embryonic fibroblasts [46].

NURR1, a ligand-independent transcriptional factor, has been studied as a therapeutic target for PD, considering its role in the transcriptional regulation of TH gene expression as well as the development and maintenance of midbrain DA neurons [47]. Kim et al. [48] recently reported that small molecules targeting the NURR1 ligand-binding domain enhanced the dual functions of Nurr1 by increasing the transcriptional activation of midbrain DA-specific genes and repressing neurotoxic proinflammatory gene expression in microglia, along with improving the motor behavioral deficits in 6-OHDA lesioned mice. Furthermore, Spathis et al. [49] reported that a NURR1: retinoid X receptor α (RXRα)-selective molecule shows symptomatic efficacy by preventing DAergic neuronal death and striatal dopaminergic denervation. However, the efficacy of these small molecules in non-motor symptoms remains to be elucidated. Based on our study, the binding affinity of NURR1 to the TH promoter in 6-OHDA-lesioned mice was elevated by SR8278 microinjection, suggesting that SR8278 microinjection has dual effects on reducing REV-ERBα activity and even enhancing NURR1 binding activity, or changing the genome-wide chromatin landscapes including the R/N motifs. It is noteworthy that the competitive action of REV-ERBα and NURR1 is important in regulating rhythmic TH expression and mood regulation. Although mood behaviors were not altered by SR8278 microinjection in VEH-treated mice at dawn, NURR1 binding affinity to the TH promoter was decreased in VEH-treated mice at dawn without changes in REV-ERBα binding affinity. The mechanisms underlying these discrepancies need to be clarified.

In addition to the ChIP assay, the time-dependent effect of SR8278 in 6-OHDA-lesioned mice could be explained by ATAC-seq differential motif analysis. Acute microinjection of SR8278 into the VTA resulted in the restoration of R/N motif enrichment in 6-OHDA-lesioned mice only at dawn, which is similar to the rescuing effect shown in the circadian mood behaviors after SR8278 treatment at dawn. SR8278 not only changed the binding activities of REV-ERBα and NURR1 on the TH promoter region but also altered the chromatin landscapes, especially the R/N binding motifs in 6-OHDA-lesioned mice. Moreover, neither 6-OHDA nor SR8278 altered E-box motif enrichment. These results indicate that the effect of SR8278 is very specific to the R/N motifs in a time-dependent manner, which supports our suggestion of chronotherapy.

It should be noted that there are limitations on currently available drugs for mood disorders in PD, although neuropsychiatric symptoms of PD are common and occur across all stages of PD [1]. DAergic therapies, including levodopa and dopamine agonists, have been used to treat mood disorders in patients with PD. Some clinical studies have reported that pramipexole, a DA receptor agonist, and in particular a D2/D3 receptor, had an antidepressant effect in patients with PD [1, 50]. However, selective serotonin reuptake inhibitors (SSRIs), which are the most common antidepressants in patients with depression, are inefficient with depression-like behaviors in a PD mouse model [39, 50]. Our studies demonstrated that SR8278 was very effective in restoring mood disorders at dawn, suggesting a potential therapeutic strategy to relieve non-motor symptoms in PD. Considering that mood-related disorders occur in a circadian manner, it is important to note that Rev-erbα might be crucial as a molecular target to improve emotional instability in PD. A single microinfusion of the REV-ERBα antagonist, SR8278, into the midbrain exerted significant antidepressant and anxiolytic effects in both 6-OHDA-lesioned mice at subjective dawn, but not at subjective dusk, thereby rescuing mood-related disorders. Chronotherapy, which considers the time of day for treatment, is increasingly important to improve the efficacy of a drug and decrease its toxicity [51]. Our study clearly showed that behavioral outcomes depend on the timing of treatment. Currently, we cannot exclude the possibility that SR8278 may also produce other transcriptional effects elsewhere in other cell types in the midbrain. In any case, we suggest that a REV-ERBα antagonist might represent a new category of drugs that regulate mood-related behaviors by modulating the temporal activities of the transcriptional repressor and activator. REV-ERBα and its ortholog REV-ERBβ showed very similar circadian expression patterns, although REV-ERBα has a greater amplitude of circadian rhythm [52]. REV-ERBα is a versatile mediator of functional circadian output in a target-dependent and tissue-specific manner [53]. REV-ERBα/β appears to be a promising target for treating diverse circadian rhythm-related disorders such as sleep disorders and metabolic diseases [54, 55].

## Conclusions

In conclusion, our data demonstrated that circadian patterns of mood regulation were disturbed with depression- and anxiety-like behaviors in a hypoDAergic state, representing human sundowning syndrome in a 6-OHDA-lesioned PD animal model. Pharmacological intervention of midbrain REV-ERBα activity rescued the circadian patterns of emotional disorders, exerting anxiolytic and antidepressant effects in a time-dependent manner. The crosstalk of nuclear receptors REV-ERB and NURR1 is important to circadian emotional control in VTA DAergic neurons of a PD model. Our findings revealed a pathophysiological mechanism of sundowning syndrome in PD, pointing to the potential of REV-ERBα as a novel therapeutic target for affective disorders related to circadian behavioral disturbances in PD.

## Supplementary Information

Below is the link to the electronic supplementary material.

Supplementary file1 (PDF 57 KB)

Supplementary file2 (PDF 57 KB)

Supplementary file3 (PDF 57 KB)

Supplementary file4 (PDF 57 KB)

Supplementary file5 (PDF 57 KB)

Supplementary file6 (PDF 57 KB)

Supplementary file7 (PDF 434 KB)

Supplementary file8 (PDF 57 KB)

Supplementary file9 (PDF 57 KB)

Supplementary file10 (PDF 57 KB)

Supplementary file11 (PDF 79 KB)

Supplementary file12 (PDF 57 KB)

Supplementary file13 (PDF 57 KB)

Supplementary file14 (PDF 26 KB)

Supplementary file15 (PDF 154 KB)

Supplementary file16 (PDF 248 KB)

Supplementary file17 (PDF 208 KB)

Supplementary file18 (PDF 41 KB)

Supplementary file19 (PDF 379 KB)

Supplementary file20 (PDF 406 KB)

Supplementary file21 (PDF 120 KB)

## Data Availability

The datasets used and/or analyzed during the current study are available from the corresponding author on reasonable request.

## Abbreviations

AD: Alzheimer’s disease
ATAC-seq: Assay for transposase-accessible chromatin with high-throughput sequencing
ChIP: Chromatin immunoprecipitation
CT: Circadian time
DAergic: Dopaminergic
DA: Dopamine
DAT: Dopamine transporter
DD: Constant dark (Dark-Dark)
EPM: Elevated plus maze
FISH: Fluorescence in situ hybridization
FST: Forced swim test
LD: Light-dark
NBRE: NGFI-B response elements
PD: Parkinson’s disease
6-OHDA: 6-Hydroxydopamine
RRE: REV-ERBs response element
SCN: Suprachiasmatic nucleus
SNpc: Substantia nigra pars compacta
SSRI: Selective serotonin reuptake inhibitors
TH: Tyrosine hydroxylase
TSS: Transcription start sites
TST: Tail suspension test
VEH: Vehicle
VTA: Ventral tegmental area
