# Correction to: Pharmacological Rescue With SR8278, A Circadian Nuclear Receptor REV-ERBα Antagonist As A Therapy for Mood Disorders In Parkinson’s Disease

**DOI:** 10.1007/s13311-022-01234-7

**Published:** 2022-05-02

**Authors:** Jeongah Kim, Inah Park, Sangwon Jang, Mijung Choi, Doyeon Kim, Woong Sun, Youngshik Choe, Ji-Woong Choi, Cheil Moon, Sung Ho Park, Han Kyoung Choe, Kyungjin Kim

**Affiliations:** 1 Department of Brain Sciences, Daegu Gyeongbuk Institute of Science and Technology (DGIST), Daegu, 42988 Korea; 2 Department of Anatomy, College of Medicine, Korea University, Seoul, Korea; 3 Program in Neurosciences, & Mental Health, The Hospital for Sick Children, Toronto, ON Canada; 4 Korea Brain Research Institute (KBRI), Daegu, Korea; 5 Department of Electrical Engineering and Computer Science, DGIST, Daegu, Korea; 6 Convergence Research Advanced Centre for Olfaction, DGIST, Daegu, Korea; 7 Department of Biological Sciences, Ulsan National Institute of Science and Technology (UNIST), Ulsan, 44919 Korea

## Correction to: Neurotherapeutics https://doi.org/10.1007/s13311-022-01215-w

The article “Pharmacological Rescue with SR8278, a Circadian Nuclear Receptor REV-ERBα Antagonist as a Therapy for Mood Disorders in Parkinson’s Disease”, written by Jeongah Kim, Inah Park, Sangwon Jang, Mijung Choi, Doyeon Kim, Woong Sun, Youngshik Choe, Ji-Woong Choi, Cheil Moon, Sung Ho Park, Han Kyoung Choe, Kyungjin Kim was originally published electronically on the publisher’s internet portal on 23 March 2022 without open access. With the author(s)’ decision to opt for Open Choice the copyright of the article changed on 28 March 2022 to © The Author(s) 2022 and the article is forthwith distributed under a Creative Commons Attribution 4.0 International License, which permits use, sharing, adaptation, distribution and reproduction in any medium or format, as long as you give appropriate credit to the original author(s) and the source, provide a link to the Creative Commons license, and indicate if changes were made. The images or other third party material in this article are included in the article's Creative Commons license, unless indicated otherwise in a credit line to the material. If material is not included in the article's Creative Commons license and your intended use is not permitted by statutory regulation or exceeds the permitted use, you will need to obtain permission directly from the copyright holder. To view a copy of this license, visit http://creativecommons.org/licenses/by/4.0/.

