# Supplementary material for: Pharmacological Rescue with SR8278, a Circadian Nuclear Receptor REV-ERBα Antagonist as a Therapy for Mood Disorders in Parkinson’s Disease
Source: Neurotherapeutics. 2022 Mar 23;19(2):592–607. doi: 10.1007/s13311-022-01215-w (PMC9226214; doi:10.1007/s13311-022-01215-w)
Supplement: Supplementary file 11 — Supplementary file11 (PDF 79 KB) [file 13311_2022_1215_MOESM11_ESM.pdf]

Supplementary Fig. 1

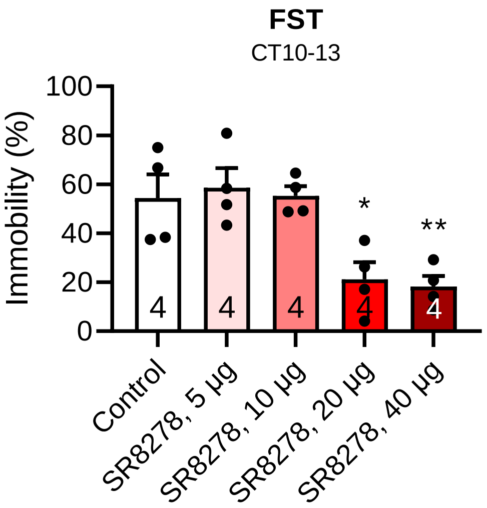

Supplementary Fig. 1 The effect of SR8278 in dose-dependent manner on depression-like behavior shown in FST in the wild-type mice at CT10-13 (one-way ANOVA, $p<0.05$). Data are presented as mean ± SEM. Sample sizes (animals) are indicated by the numbers inside bars. Bonferroni corrected post-hoc comparisons are indicated by *$p<0.05$ and **$p<0.01$.
