# Supplementary material for: Pharmacological Rescue with SR8278, a Circadian Nuclear Receptor REV-ERBα Antagonist as a Therapy for Mood Disorders in Parkinson’s Disease
Source: Neurotherapeutics. 2022 Mar 23;19(2):592–607. doi: 10.1007/s13311-022-01215-w (PMC9226214; doi:10.1007/s13311-022-01215-w)
Supplement: Supplementary file 14 — Supplementary file14 (PDF 26 KB) [file 13311_2022_1215_MOESM14_ESM.pdf]

Supplementary Fig. 2

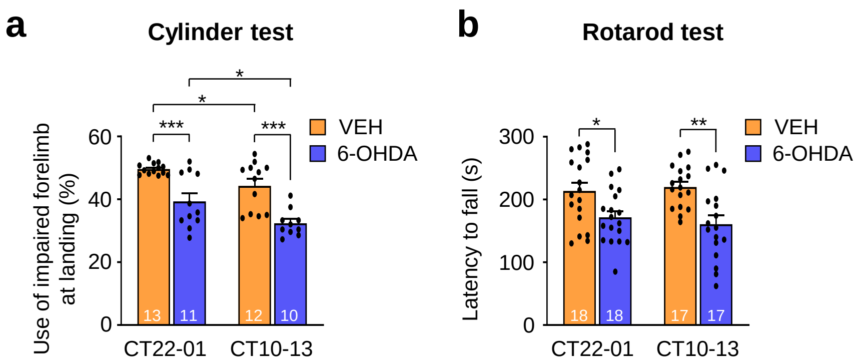

Supplementary Fig. 2 The effect of 6-OHDA lesion to motor behaviors. Motor impairment behaviors in the cylinder (**a**) and rotarod (**b**) tests at the indicated times for VEH- and 6-OHDA-injected mice (cylinder test: two-way ANOVA, $p<0.0001$ for 6-OHDA-lesion, $p=0.0016$ for time, $p=0.6634$ for interaction) (rotarod test: two-way ANOVA, $p<0.0001$ for 6-OHDA-lesion, $p=0.8436$ for time, $p=0.4525$ for interaction). Data are presented as mean ± SEM. Sample sizes (animals) are indicated by the numbers inside bars. Newman-keuls corrected post-hoc comparisons are indicated by *$p<0.05$, **$p<0.01$ and ***$p<0.001$
