# Supplementary material for: Pharmacological Rescue with SR8278, a Circadian Nuclear Receptor REV-ERBα Antagonist as a Therapy for Mood Disorders in Parkinson’s Disease
Source: Neurotherapeutics. 2022 Mar 23;19(2):592–607. doi: 10.1007/s13311-022-01215-w (PMC9226214; doi:10.1007/s13311-022-01215-w)
Supplement: Supplementary file 15 — Supplementary file15 (PDF 154 KB) [file 13311_2022_1215_MOESM15_ESM.pdf]

Supplementary Fig. 3

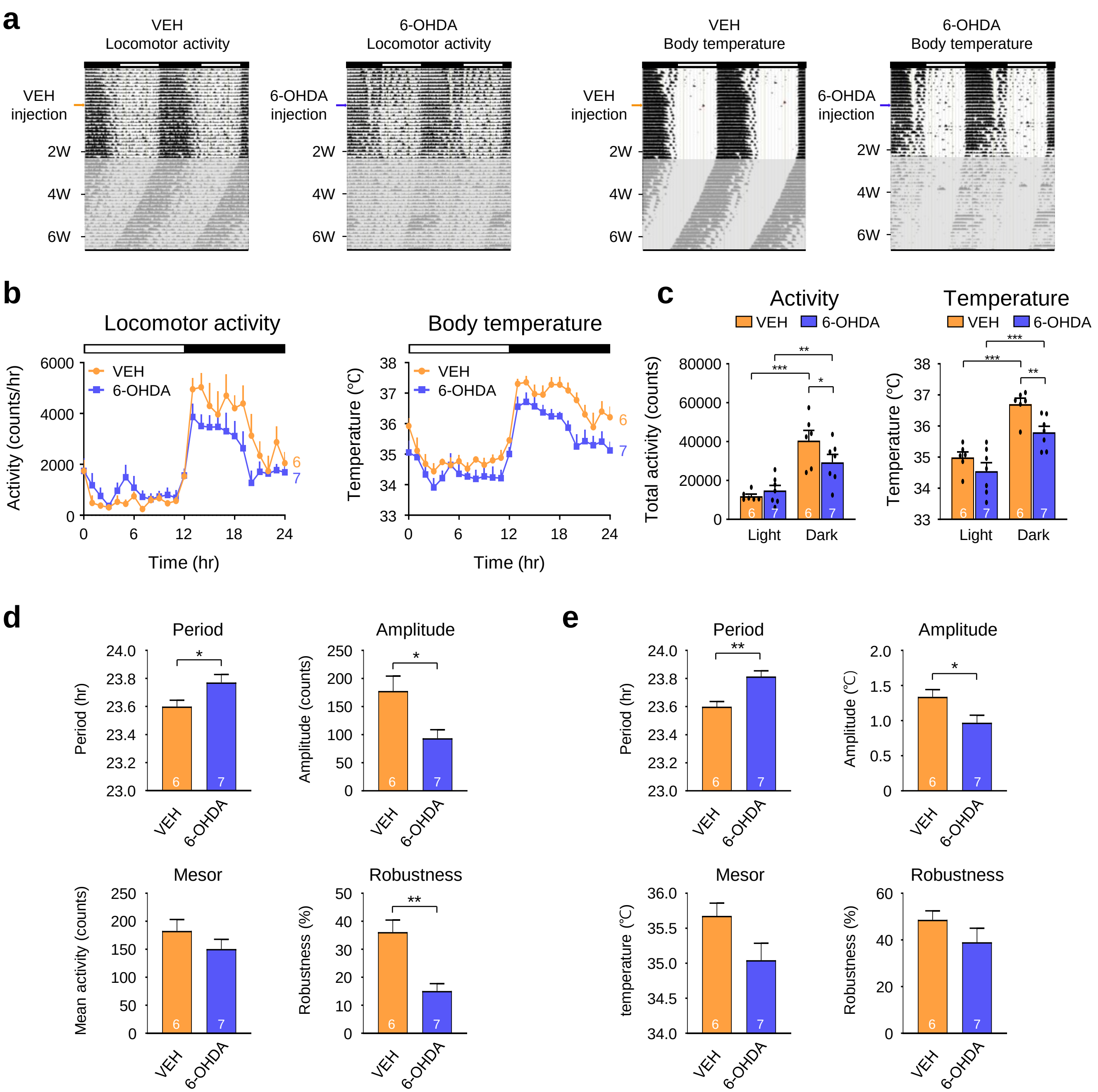

Supplementary Fig. 3 6-OHDA treatment alters circadian behavior. (a) Representative images of actogram and body temperature rhythm illustrating the effect of single injections of VEH and 6-OHDA on circadian behavior. C57BL6 mice were initially maintained on a 12h:12h L:D cycle and animals were injected with VEH or 6-OHDA. After 2 W on L:D, mice were kept in constant darkness (D:D). (**b**) Daily rhythm of locomotor activity and body temperature in condition with L:D cycles at 2 W following VEH or 6-OHDA injection (locomotor activity: two-way RM-ANOVA, $p<0.0001$ for time, $p=0.3365$ for 6-OHDA lesion, $p=0.0102$ for interaction) (body temperature: two-way RM-ANOVA, $p<0.0001$ for time, $p=0.0474$ for 6-OHDA lesion, $p=0.1201$ for interaction). Sample sizes (animals) are indicated by the numbers on the graphs. (**c**) Total activity and average body temperature at light and dark period at 2 W following VEH or 6-OHDA injection in condition with L:D cycle (locomotor activity: two-way ANOVA, $p=0.2631$ for 6-OHDA-lesion, $p<0.0001$ for time, $p=0.0626$ for interaction) (body temperature: two-way ANOVA, $p=0.0057$ for 6-OHDA-lesion, $p<0.0001$ for time, $p=0.3034$ for interaction). Newman-keuls post-hoc comparisons are indicated by *$p<0.05$, **$p<0.01$, ***$p<0.001$ (**d**) Analysis of locomotor activity in mice kept under constant darkness using cosinor anlaysis program (student t-test; $p=0.0407$ for period, $p=0.0155$ for amplitude, $p=0.2526$ for mesor, $p=0.0012$ for robustness. (**e**) Analysis of body temperature in mice kept under constant darkness using cosinor analysis program (student t-test; $p=0.0026$ for period, $p=0.0316$ for amplitude, $p=0.0677$ for mesor, $p=0.2222$ for robustness). Sample sizes (animals) are indicated by the numbers inside bars. The data were presented as mean ± SEM
