# Supplementary material for: Pharmacological Rescue with SR8278, a Circadian Nuclear Receptor REV-ERBα Antagonist as a Therapy for Mood Disorders in Parkinson’s Disease
Source: Neurotherapeutics. 2022 Mar 23;19(2):592–607. doi: 10.1007/s13311-022-01215-w (PMC9226214; doi:10.1007/s13311-022-01215-w)
Supplement: Supplementary file 16 — Supplementary file16 (PDF 248 KB) [file 13311_2022_1215_MOESM16_ESM.pdf]

Supplementary Fig. 4

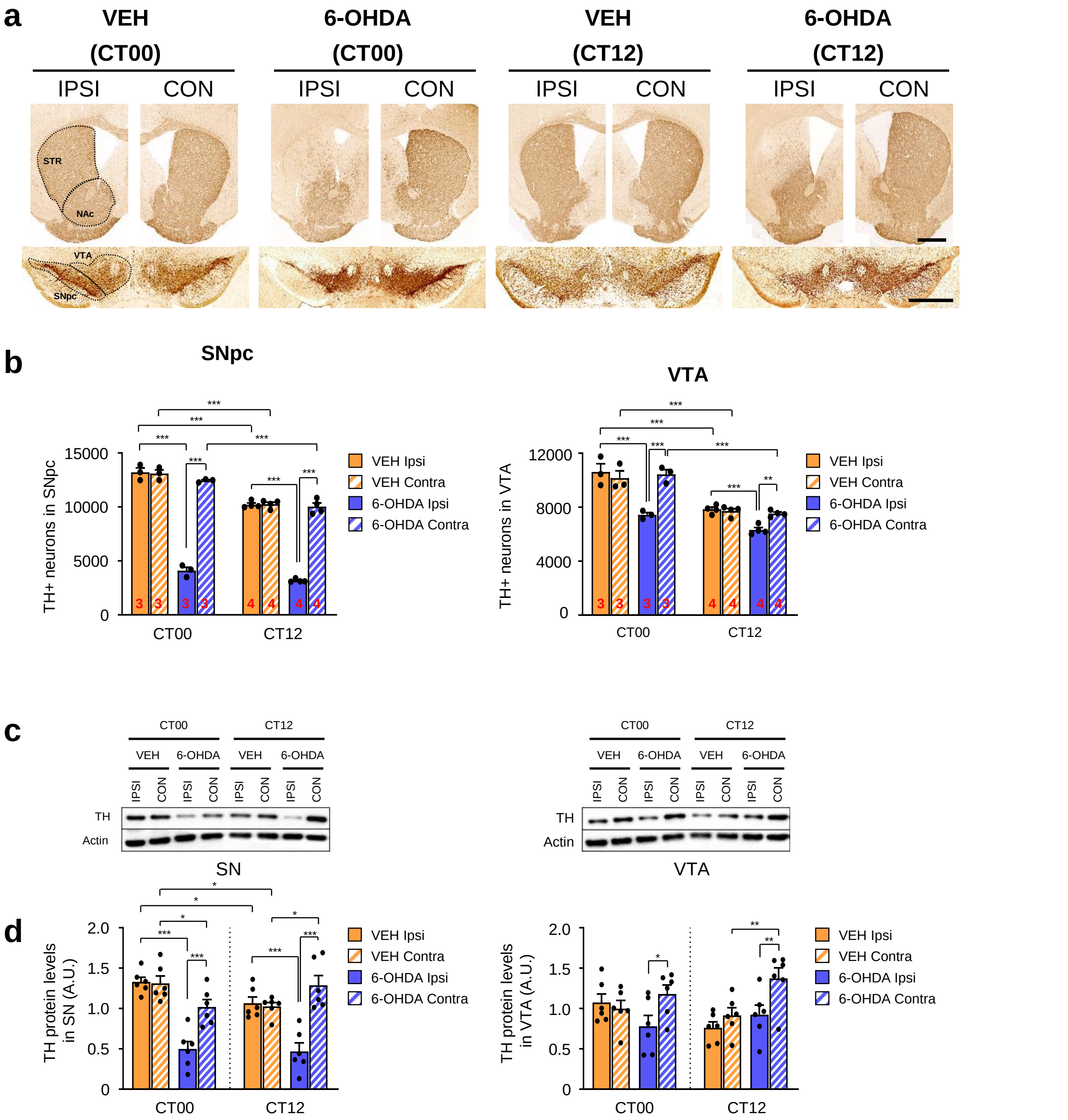

Supplementary Fig. 4 Tyrosine hydroxylase expression of DAergic neurons in the SNpc and VTA of VEH- and 6-OHDA-injected mice. (a) Coronal brain sections containing ipsilateral (IPSI) and contralateral (CON) sides of the striatum (STR) and nucleus accumbens (NAc) of VEH- and 6-OHDA-lesioned mice were immunolabeled with tyrosine hydroxylase (TH). Scale bar = 500 μm. TH labeling of SNpc and VTA. Scale bar = 200 μm. (b) Stereological TH-positive cell counting in SNpc and VTA using 6 slices per animal with Stereo Investigator (SNpc: two-way ANOVA, $p<0.0001$ for time, $p<0.0001$ for group, $p=0.0022$ for interaction) (VTA: two-way ANOVA, $p<0.0001$ for time, $p<0.0001$ for group, $p=0.0280$ for interaction). Sample sizes (animals) are indicated by the numbers inside bars. Fisher's LSD post-hoc comparisons are indicated by **$p<0.01$ and ***$p<0.001$. (**c**) Representative immunoblot images showing TH protein expression in the SNpc and VTA of VEH- and 6-OHDA-lesioned mice at CT00 and CT12. (**d**) TH protein levels in SN and VTA. After densitometric analysis, TH protein levels were normalized with actin levels (SN: two-way ANOVA, $p<0.0001$ for group, $p=0.2291$ for time, $p=0.0115$ for interaction, n=6 per each group) (VTA: $p=0.0022$ for group, $p=0.8567$ for time, $p=0.1048$ for interaction, n=6 per each group). Fisher's LSD post-hoc comparisons are indicated by *$p<0.05$, **$p<0.01$ and ***$p<0.001$. Data were presented as mean ± SEM
