# Supplementary material for: Pharmacological Rescue with SR8278, a Circadian Nuclear Receptor REV-ERBα Antagonist as a Therapy for Mood Disorders in Parkinson’s Disease
Source: Neurotherapeutics. 2022 Mar 23;19(2):592–607. doi: 10.1007/s13311-022-01215-w (PMC9226214; doi:10.1007/s13311-022-01215-w)
Supplement: Supplementary file 17 — Supplementary file17 (PDF 208 KB) [file 13311_2022_1215_MOESM17_ESM.pdf]

Supplementary Fig. 5

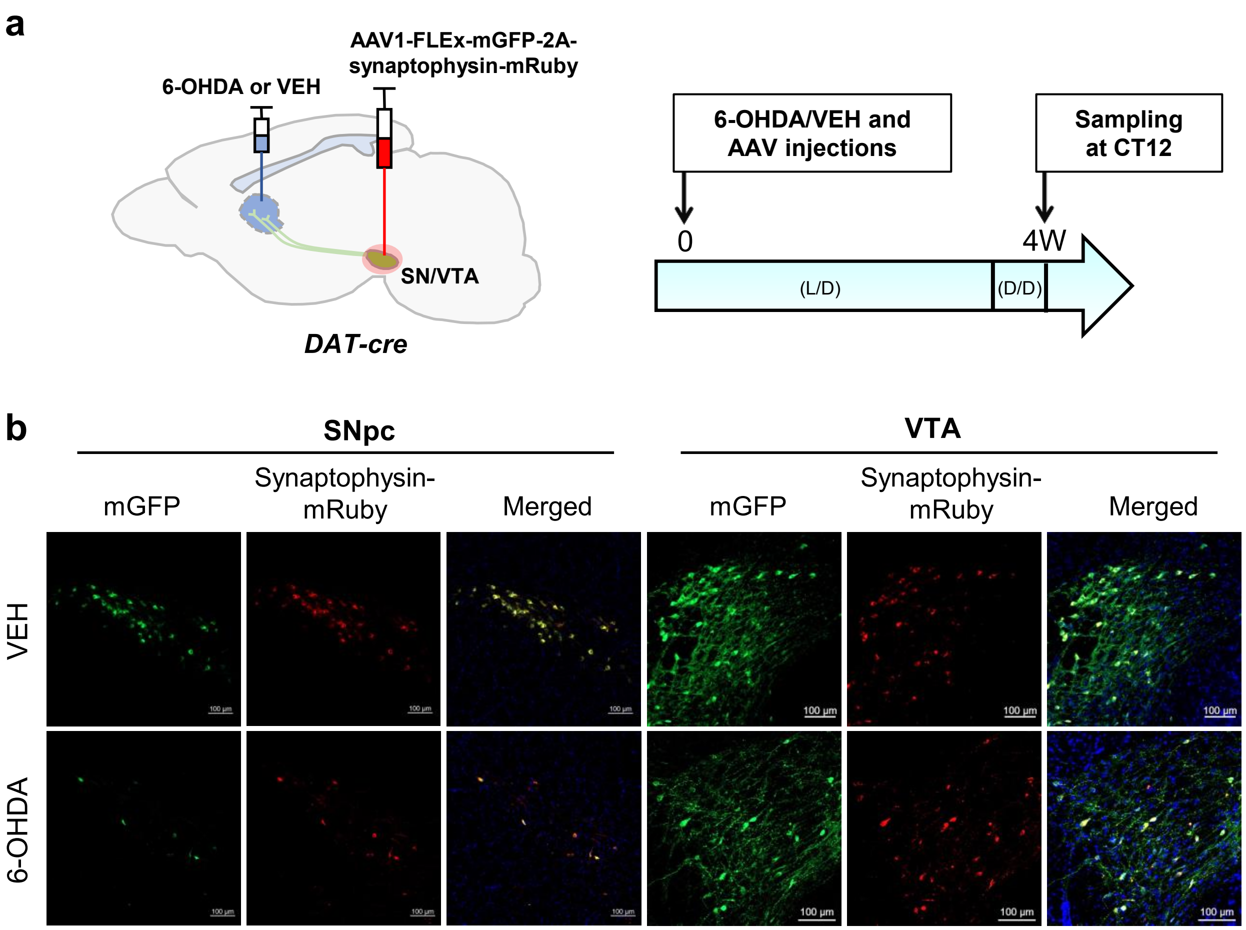

Supplementary Fig. 5 (a) Experimental scheme to confirm the DAergic neuronal loss in 6-OHDA-lesioned mice with TH-independent marker. 6-OHDA or vehicle was microinjected into dorsal striatum, and AAV1-hSyn-FLEx-mGFP-2A-Synaptophysin-mRuby was simultaneously microinjected into the SNpc/VTA of DAT-cre knock-in mice**.** After 4 weeks post injection, mouse brains were acquired at circadian time 12 (CT12). (b) Coronal brain sections containing ipsilateral (IPSI) sides of the SNpc/VTA of VEH- or 6-OHDA-lesioned DAT-cre mice with mGFP-labelled DAergic neurons and mRuby-tagged synaptophysin were presented. Scale bar = 100 μm.
