# Supplementary material for: Pharmacological Rescue with SR8278, a Circadian Nuclear Receptor REV-ERBα Antagonist as a Therapy for Mood Disorders in Parkinson’s Disease
Source: Neurotherapeutics. 2022 Mar 23;19(2):592–607. doi: 10.1007/s13311-022-01215-w (PMC9226214; doi:10.1007/s13311-022-01215-w)
Supplement: Supplementary file 18 — Supplementary file18 (PDF 41 KB) [file 13311_2022_1215_MOESM18_ESM.pdf]

Supplementary Fig. 6

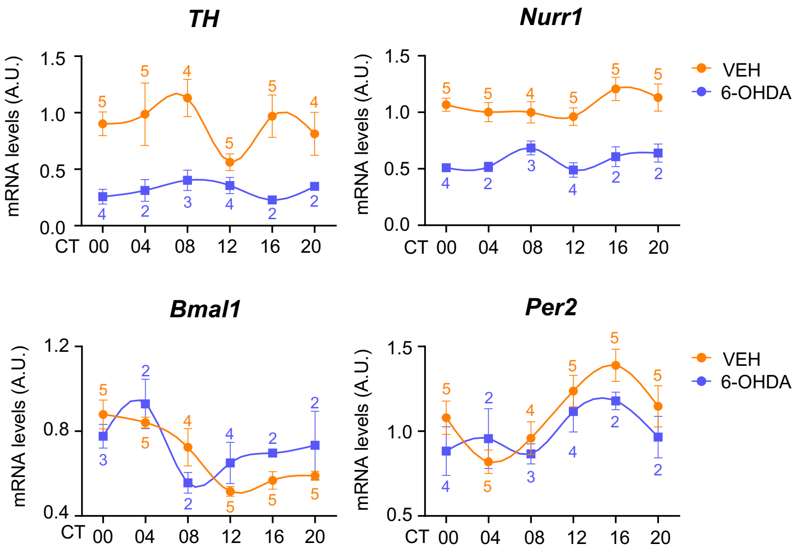

Supplementary Fig. 6 The effect of 6-OHDA lesion to transcription levels of DA-related genes and circadian clock genes. Circadian mRNA expression by RT-qPCR in the midbrain lysate. Sample sizes (animals) are indicated by the numbers on the graphs. Group differences were evaluated by two-way ANOVA (*TH*: p<0.0001 for 6-OHDA-lesion, p=0.6073 for time, p=0.5654 for interaction; *Nurr1*: p<0.0001 for 6-OHDA-lesion, p=0.3674 for time, p=0.7644 for interaction; *Bmal1*: p=0.3486 for 6-OHDA lesion, p=0.0002 for time, p=0.1068 for interaction; *Per2*: p=0.1156 for 6-OHDA lesion, p=0.0156 for time, p=0.7509 for interaction). The data were presented as mean ± SEM
