# Supplementary material for: Pharmacological Rescue with SR8278, a Circadian Nuclear Receptor REV-ERBα Antagonist as a Therapy for Mood Disorders in Parkinson’s Disease
Source: Neurotherapeutics. 2022 Mar 23;19(2):592–607. doi: 10.1007/s13311-022-01215-w (PMC9226214; doi:10.1007/s13311-022-01215-w)
Supplement: Supplementary file 19 — Supplementary file19 (PDF 379 KB) [file 13311_2022_1215_MOESM19_ESM.pdf]

Supplementary Fig. 7

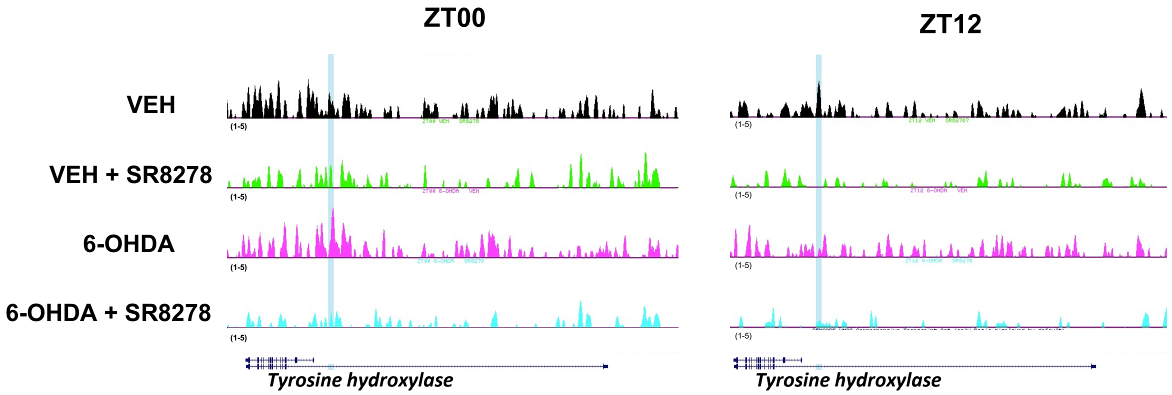

Supplementary Fig. 7 Chromatin-accessibility is changed with circadian time points in VTA brain regions. Visualization of ATAC-seq peak signals in TH genomic location showing the greater number of ATAC-seq peaks at ZT00 compares to that of ZT12. The R/N binding sites in TH promoter region were indicated with the blue bars. For each ATAC-seq track, VTA from five mice was pooled for sequencing (n=5 per pooled sample)
