# Supplementary material for: Pharmacological Rescue with SR8278, a Circadian Nuclear Receptor REV-ERBα Antagonist as a Therapy for Mood Disorders in Parkinson’s Disease
Source: Neurotherapeutics. 2022 Mar 23;19(2):592–607. doi: 10.1007/s13311-022-01215-w (PMC9226214; doi:10.1007/s13311-022-01215-w)
Supplement: Supplementary file 20 — Supplementary file20 (PDF 406 KB) [file 13311_2022_1215_MOESM20_ESM.pdf]

Supplementary Fig. 8

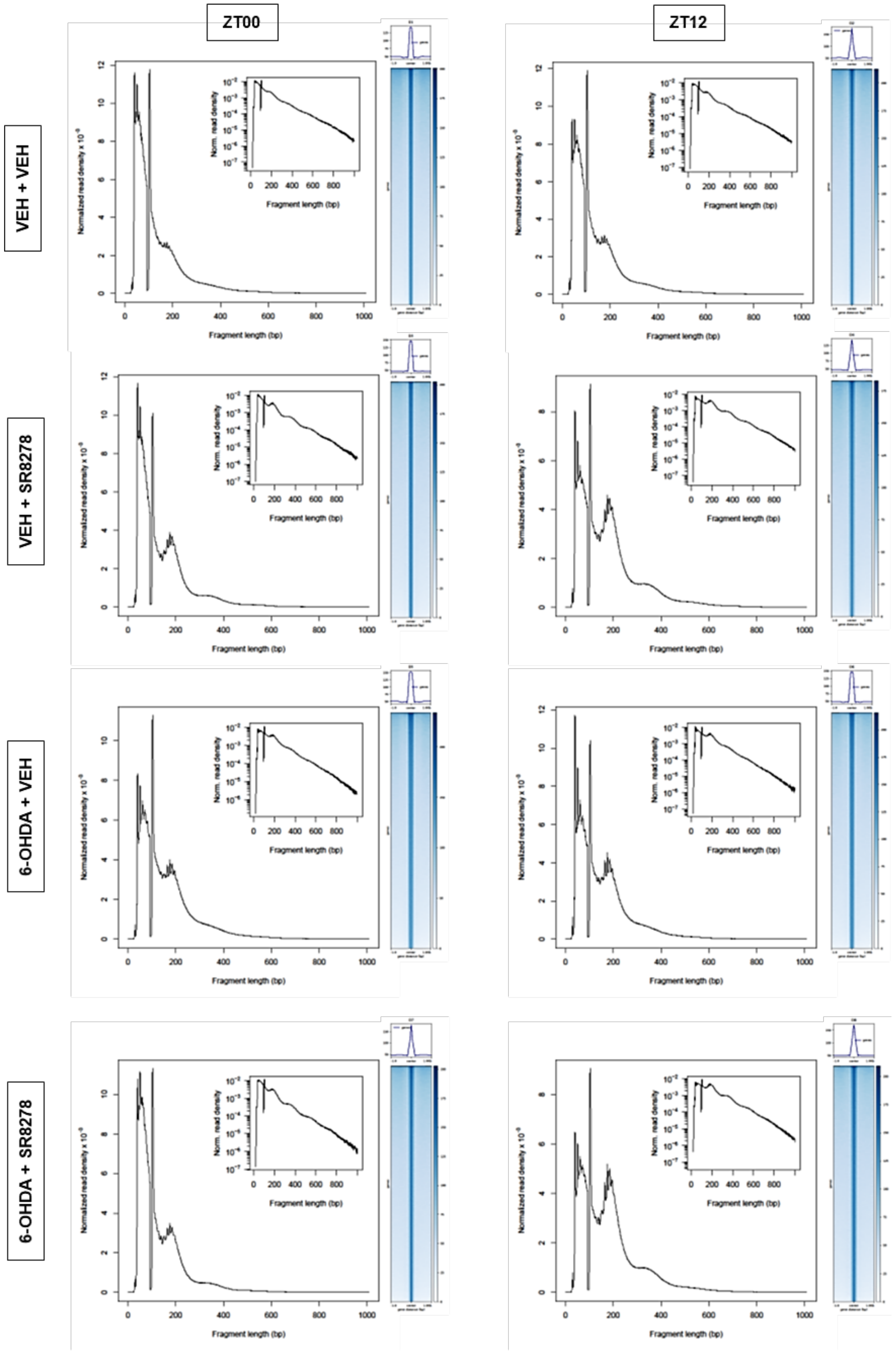

Supplementary Fig. 8 Fragment distributions of each library for the ATAC-seq quality control. The fragment distributions for each library of ATAC-seq datasets were assessed using the ATACseqQC and Heatmaps of ATAC-seq accessibility of transcription start site (center) using deepTools. The genomics features are defined 1Kb from the center
