# Supplementary material for: Pharmacological Rescue with SR8278, a Circadian Nuclear Receptor REV-ERBα Antagonist as a Therapy for Mood Disorders in Parkinson’s Disease
Source: Neurotherapeutics. 2022 Mar 23;19(2):592–607. doi: 10.1007/s13311-022-01215-w (PMC9226214; doi:10.1007/s13311-022-01215-w)
Supplement: Supplementary file 21 — Supplementary file21 (PDF 120 KB) [file 13311_2022_1215_MOESM21_ESM.pdf]

Supplementary Fig. 9

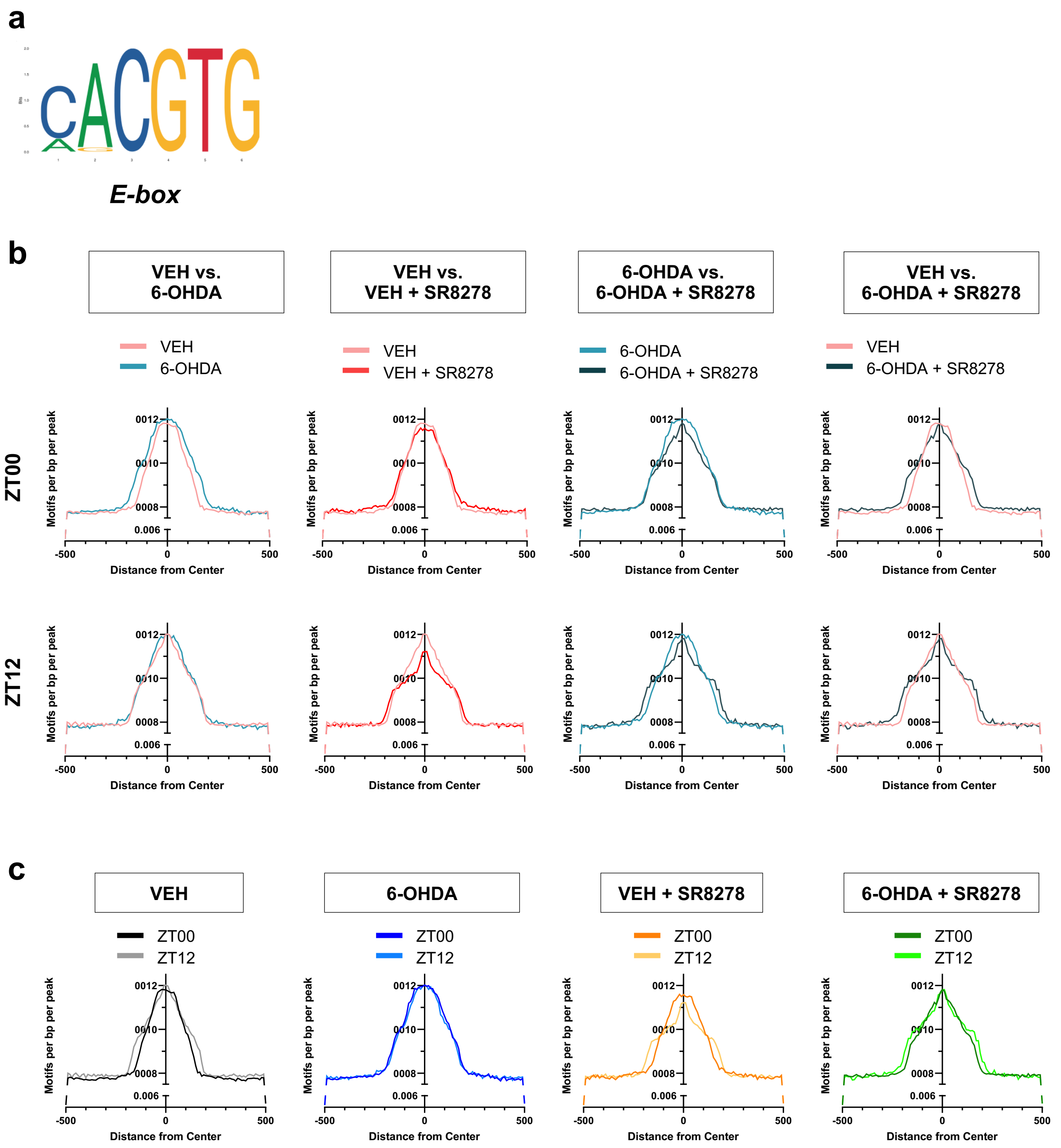

Supplementary Fig. 9 6-OHDA and SR8278 treatments to VTA did not alter the enrichments of E-box motifs. (**a**) A sequence logo for E-box motif is depicted. (**b**) Probability of E-box motifs was calculated using HOMER from called peaks and compared the motif enrichment results between indicated experimental groups. (**c**) Enrichment of E-box motifs was analyzed and presented indicated time points. For each ATAC-seq data analysis, VTA regions were pooled from five individual mice (n=5, pooled)
